# The GATA-like transcription factor Gat201 determines alkaline-restricted growth in *Cryptococcus neoformans*

**DOI:** 10.1128/msphere.00191-25

**Published:** 2025-06-04

**Authors:** Elizabeth S. Hughes, Laura R. Tuck, Zhenzhen He, Elizabeth R. Ballou, Edward W. J. Wallace

**Affiliations:** 1 Institute for Cell Biology, and Centre for Engineering Biology, School of Biological Sciences, The University of Edinburgh, Edinburgh, United Kingdom; 2 MRC Centre for Medical Mycology, The University of Exeter 601337, Exeter, United Kingdom; Virginia-Maryland College of Veterinary Medicine, Blacksburg, Virginia, USA

**Keywords:** *Cryptococcus neoformans*, transcriptional regulation, pH

## Abstract

The fungus *Cryptococcus neoformans* is an opportunistic human pathogen that causes fatal meningitis through uncontrolled proliferation in host tissues. Evasion of host defenses relies on a protective polysaccharide capsule, regulated, in part, by the GATA-like transcription factor Gat201. Gat201 additionally contributes to virulence through capsule-independent mechanisms. Here, we show that Gat201 affects the proliferation of *C. neoformans*: in RPMI-1640 cell culture media at an alkaline pH that restricts wild-type cell growth, *gat201*∆ strains show increased budding, growth, and viability. RNA-seq analysis shows that Gat201 pathway genes, including co-factors *GAT204* and LIV3, are rapidly activated within minutes of inoculating *C. neoformans* in RPMI media, and strains mutated for *GAT204* and, to a lesser extent, *LIV3* also show improved growth. The effect of Gat201 on growth is pH-dependent: *gat201*∆ cells grow better than wild-type cells at high pH but worse than wild-type cells at neutral pH, in otherwise identical media. Together, this identifies the Gat201 pathway as an alkaline-responsive regulator of proliferation: Gat201 appears to govern an environment-dependent trade-off between proliferation and production of the defensive capsule. Furthermore, evolutionary analysis shows that Gat201 is in a subfamily of GATA-like transcription factors that is conserved within diverse fungi but absent in model yeasts. Together, our findings urge improved understanding of proliferation in diverse environmental niches in order to understand the mechanistic basis of fungal pathogenesis.

**IMPORTANCE:**

Infectious microorganisms must adapt to differences between external and host environments in order to colonize and cause disease. *Cryptococcus neoformans* is an encapsulated fungal pathogen that can infect human airways and travel to the brain to cause life-threatening meningitis. The airway is a dynamic environment characterized by nutrient limitation, high temperature (37°C), CO_2_, and transiently high pH (>8.5). In both the lung and brain, fungal proliferation through budding is a major driver of pathogenesis; however, the regulators of *Cryptococcus* proliferation are poorly understood and distinct from other model yeasts. In this work, we explore how *Cryptococcus* adapts to shifting environments and identify that the transcription factor Gat201, known to regulate capsule production, negatively regulates proliferation under alkaline conditions. Our findings highlight the need for improved understanding of proliferation/adaptation and its regulation in non-model systems.

## INTRODUCTION

*Cryptococcus neoformans* is an environmental saprophyte and a critical priority human fungal pathogen (1) that is a major cause of death in human immunodeficiency virus (HIV)-positive individuals (2, 3). *C. neoformans* is a basidiomycete fungus that is associated worldwide with bird guano and arboreal habitats (4–6) but can become an opportunistic pathogen following inhalation of airborne spores or desiccated yeast cells into mammalian airways (7–10). These infectious particles are initially metabolically inactive and must reactivate proliferation and simultaneously escape the immune system to infect host airways (8, 11–13). Proliferation of *Cryptococcus* in the upper airways or lungs can be followed by dissemination through other host niches, including the central nervous system, causing fatal meningitis. In the host, reactivated *Cryptococcus* cells produce a protective polysaccharide capsule that promotes immune evasion and is essential to *Cryptococcus* survival and dissemination in the host (14, 15) but is dispensable for fungal proliferation *in vitro* (16). Thus, both adaptation to diverse environmental niches and immune evasion are crucial for cryptococcal virulence (15, 17–20).

We set out to understand the regulatory pathways involved in *C. neoformans’* adaptation to environmental niches, using *in vitro* cell culture media that mimic some aspects of human airway surfaces. As the human airway surface becomes alkaline during each inhaled breath (21), we investigated which genes are induced in *C. neoformans* stationary phase yeast cells as they reactivate after inoculation into RPMI-1640 media at an alkaline pH. Our RNA-seq revealed strong induction of a virulence-related pathway in these conditions: the Gat201 pathway.

The GATA-like zinc finger transcription factor Gat201 is a key regulator of virulence that acts through capsule-dependent (22) and capsule-independent mechanisms (23). *C. neoformans* strains with *GAT201* deleted have reduced capsule size (24–26) and are also more readily taken up by mammalian macrophages, independent of capsule production (23). The genes targeted by Gat201 have been mapped by RNA-seq and ChIP-seq (23, 27, including *GAT204* and *LIV3,* encoding transcriptional co-factors of Gat201 that themselves target an overlapping set of genes. However, the nature of the Gat201 pathway’s capsule-independent contributions to virulence remains unexplained. One challenge is that *gat201Δ* exhibits weak phenotypes in standard microbiological growth conditions (24), making it difficult to probe Gat201 virulence-relevant regulation *in vitro*.

Here, we report that the Gat201 pathway appears to govern an environment-dependent choice between proliferation and capsule formation: wild-type cells inoculated into RPMI media at an alkaline pH proliferate poorly and make capsules, but the deletion of *GAT201* dramatically improves proliferation and long-term viability but decreases capsule formation. This suggests that poor growth in our *in vitro* conditions is a consequence of regulated gene expression, controlled by the transcription factor Gat201, rather than a physiological response to nutrient starvation. We identify *GAT201*-dependent transcriptional signatures of this phenotype and demonstrate that the Gat201 pathway is activated to suppress proliferation under alkaline conditions only, independently of serum and of cyclic AMP. Our findings that Gat201 regulates proliferation in a pH-dependent manner, and our analysis of Gat201 homologs in other fungal pathogens, suggest that Gat201 is part of a conserved pathway involved in regulating fungal growth in response to environmental stimuli.

## RESULTS

### *C. neoformans* rapidly induces media-specific growth programs upon reactivation from stationary phase

To better understand how metabolically inactive *C. neoformans* yeast cells adapt to the initial interaction with the host, we first modeled the switch from stationary cultures (5 days of growth in YPD) to different growth conditions, including nutrient limitation, temperature, and low-oxygen conditions. We performed a hypothesis-generating “range-finding” experiment to gain insight into the initial transcriptional responses that characterized this transition. We compared reactivation in a nutrient-rich microbial growth medium (YPD) to the cell culture medium (RPMI-1640 + 10% heat-inactivated fetal calf serum; abbreviated RPMI+ serum) at two different temperatures (25°C or 37°C), 60 rpm, and measured RNA abundance by RNA-seq at 10, 30, 60, and 120 min after inoculation (Fig. S1).

Overall, this range-finding experiment showed that reactivating cryptococcal cells rapidly activate transcription and biosynthetic pathways, including the Gat201 pathway (see supplemental results and Fig. S1 to S3). We observed over 10-fold induction of *GAT201* and its co-factors *GAT204* and *LIV3* in RPMI+ serum. By microscopy, we additionally observed that within 2.5 h in RPMI+ serum at 37°C, cells make a polysaccharide capsule that is associated with cellular defense but do not start budding (Fig. S1). By contrast, in YPD media, cells proliferate by producing buds within 2.5 h of inoculation (Fig. S1). Transcriptional analysis revealed that YPD-reactivated cells induce both protein synthesis and cell cycle progression but do not induce the Gat201 pathway (Fig. S1). These observations raised the hypothesis that the Gat201 pathway is specifically activated in RPMI media and could be involved in a condition-dependent decision between proliferation and defense.

### *GAT201* regulates proliferation and viability

**Fig 1:**
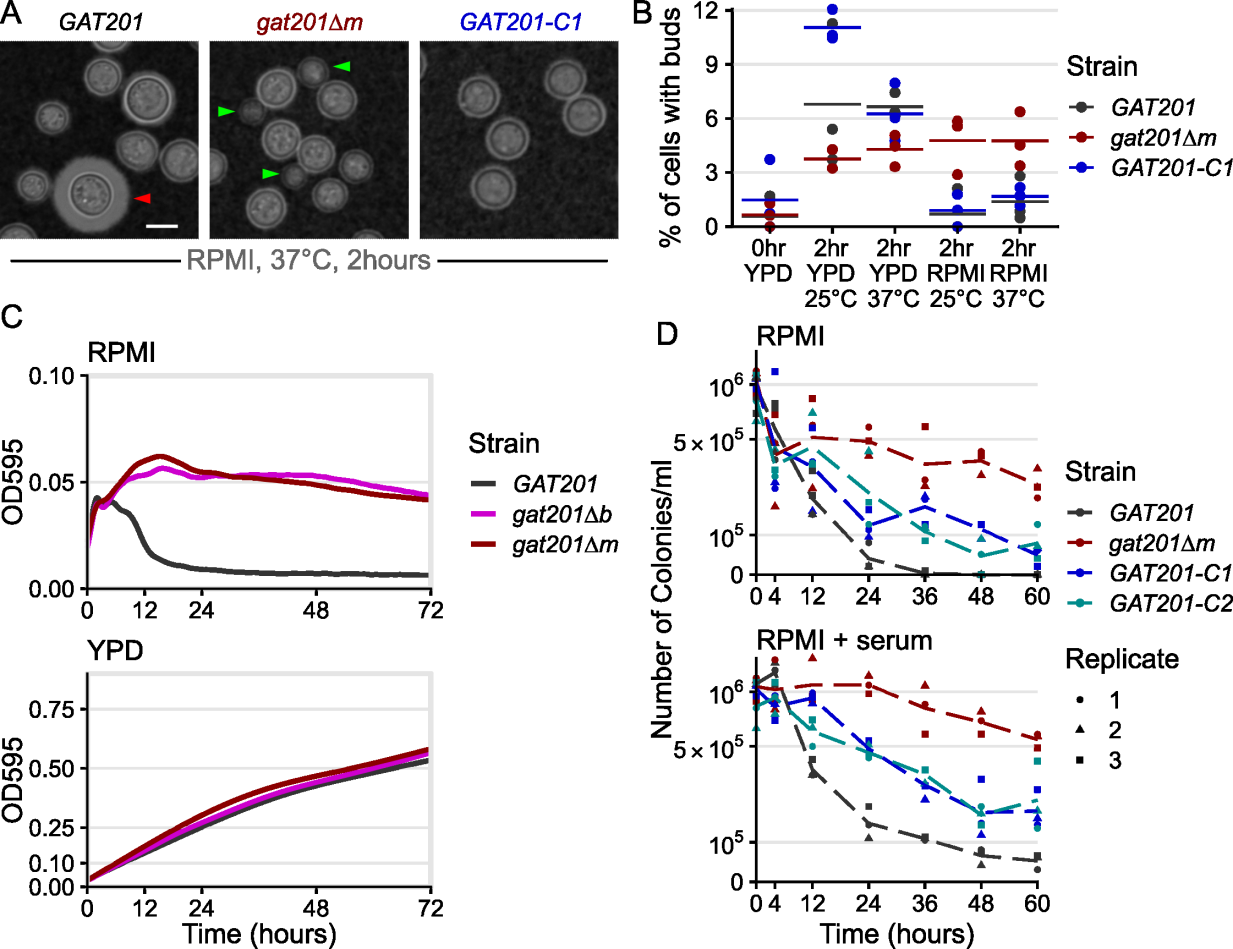
*GAT201* represses the proliferation and viability of *C. neoformans* during reactivation in RPMI medium. (A) *GAT201* promotes capsule biosynthesis and represses budding in RPMI-1640 medium (without serum) at 37°C 2 h after inoculation. Micrographs show *GAT201* (H99 wild-type), *gat201*∆*m*, and complemented *GAT201-C1* strains, stained with India Ink, capsule highlighted with red arrow, and buds highlighted with green arrows. *GAT201-C1* complements the budding phenotype but does not clearly complement the capsule phenotype. (B) Quantification of budding index at 2 h (% budded cells) shows that *gat201*∆*m* cells reactivate to produce buds in RPMI (*n* = >100 cells per replicate, with three biological replicates per condition). Panels A and B are taken from the same experiment, and larger sets of representative cells are shown in Fig. S4. (C) *GAT201* (H99) cell populations reactivating in RPMI show a fall in density after 10 h of growth, which is absent in *gat201*∆ strains and absent during growth in rich YPD media. Growth curves of optical density at 595 nm (OD_595_) were collected via plate reader from seven biological replicates, three technical replicates each, at 37°C. Note the different y-axis limits in the subpanels, reflecting higher final OD in rich media. (D) *GAT201* (H99) cells reactivating in RPMI or RPMI+ serum show a decline in viability after 12–24 h, which is absent in *gat201*∆ and partially restored by complementing *GAT201*. The decline in viability is more severe in RPMI without serum than it is in RPMI with serum. Colony-forming units per milliliter of culture were measured by serial dilution on plates, in three biological replicates; individual replicates are plotted as dots with a dashed line connecting the medians.

To test the hypothesis that *GAT201* impacts reactivation, we used two independently generated mutants: *gat201*∆*m* is a complete deletion of the reading frame from start codon to stop codon from the Madhani collection (22), and *gat201*∆*b* is a disruption of the protein from the Bahn collection (24). We also made two independent complemented strains in the *gat201*∆*m* background, *GAT201-C1* and *GAT201-C2*. We phenotyped these strains in a simplified assay: shift from a 5-day YPD culture to RPMI at two different temperatures, conditions under which we observed similar proliferation phenotypes by wild-type cells to our range-finding experiment (Fig. 1A and B).

Our mutant analysis revealed that *GAT201* represses proliferation and viability during reactivation in RPMI medium. First, we observed growth and capsule production 2 h after inoculation in RPMI at 37°C. Wild-type *GAT201* strains produce a capsule and have few visible buds, whereas *gat201*∆ strains have visible buds and small capsules (Fig. 1A; Fig. S4). This is consistent with previous work showing *gat201*∆ to be defective in capsule (22, 25, 26). There is no visible difference in phenotype during growth in YPD (Fig. S4). Genetic complementation of *GAT201* represses bud formation in RPMI. We observed partial restoration of the capsule, likely due to lower expression of *GAT201* mRNA in complemented strains, measured by RT-qPCR as roughly ~10× lower than that in wild-type (Fig. S5). We also confirmed by RT-qPCR that *GAT201* complementation restores the downstream expression of Gat201 targets, *GAT204* and *LIV3* (Fig. S5).

To quantify this defect, we measured budding at 2 h post-reactivation. As before, growth condition impacted budding, with 6–10% of wild-type cells producing visible buds in YPD media, but only 1–2% in RPMI media (Fig. 1B). *GAT201* also quantitatively affects budding: deletion of *GAT201* increases budding in RPMI, with roughly 5% of *gat201*∆ cells budding within 2 h, a more than 2-fold increase. Genetic complementation of *GAT201* reduces bud formation to near-wild-type percentage in RPMI (Fig. 1B). This 2 h time point was selected because it is sufficient time for *C. neoformans* yeast to complete only a single cell cycle (28), allowing detection of only the first bud from mother cells without the risk of measuring repeated budding.

Second, we set out to study the longer-term impacts of *GAT201* on growth, and surprisingly, we observed that *GAT201* suppresses growth in alkalinized RPMI medium (Fig. 1C). These experiments were conducted in an RPMI formulation buffered with sodium bicarbonate and grown under aerobic conditions so that the pH rose to become alkaline over the course of the experiment. Although wild-type cells in RPMI medium did initially increase in optical density for 4 h, density then rapidly declined within 10 h (Fig. 1C). In contrast, *gat201*∆ cultures for both mutants continually increased in cell density over 12 h and maintained a higher OD_595_ of 0.05 over 3 days (Fig. 1C). This effect is media-specific: *GAT201* cells and *gat201*∆ mutants grew similarly in rich media, reaching a similar OD_595_ of around 0.5 after 3 days (Fig. 1C). Together, these data suggested that Gat201 suppresses growth in alkalinized conditions.

Finally, to determine if the *GAT201*-dependent reduction in cell density in RPMI represented a loss of viability, we quantified colony-forming units (CFUs) from cells grown in RPMI at 37°C over 48 h (Fig. 1D). All cultures started with approximately the same number of stationary cells per milliliter (1 × 10^6^, OD_595_ = 0.1). By 24 h, we observed a 25-fold reduction (to 4 × 10^4^) in the wild-type cultures, compared with a 2-fold (5 × 10^5^ cells/mL) reduction in the number of viable cells/mL in the *gat201*∆ mutant. By 36 h, *GAT201* wild-type viability was 100-fold less (1 × 10^4^ cells/mL), whereas viability in the *gat201*∆ mutant strain had decreased by only 3-fold (3.7 × 10^5^ cells/mL). *GAT201* viability dropped to zero by 48 h. In contrast, the *gat201*∆ mutant strain remained viable for up to 60 h post-inoculation, with a 4-fold decrease (2.7 × 10^5^ cells/mL). Genetic complementation of *GAT201* reduced viability, although not to wild-type levels. Importantly, the impact of *GAT201* on viability is independent of serum (Fig. 1D). Together, these data suggested that Gat201 limits cellular viability in alkalinized conditions.

### The Gat201 transcriptional pathway is required for alkaline-restricted growth

Given the differential viability of wild-type and *gat201*∆ cells in RPMI media, we investigated the transcriptional pathways that might be responsible for these phenotypes. By RNA-seq, we observed that deletion of *GAT201* indeed prevents activation of previously established Gat201 targets (Fig. S10). We measured two independent mutants (*gat201*∆*m* and *gat201*∆*b*) and two congenic wild-type *GAT201* strains (KN99 MATa and MATalpha) (29), each measured in two biological replicates. Thus, there are effectively four biological replicates per relevant genotype. We measured RNA abundance across four time points (0, 30, 120, and 240 min), in RPMI-1640 media both with and without serum. As described in detail in the supplement, the results were highly reproducible across strains by principal component analysis (PCA): time after inoculation was the dominant driver of transcriptional differences during reactivation in RPMI, both with and without serum (Fig. S6 to S9).

Several hundred genes were found to be differentially expressed dependent on *GAT201* across the time course, with more differential expression at later time points (Fig. S12). In agreement with previous functional genomics studies (27), the majority of *GAT201*-dependent genes are direct targets of Gat201 as reported by ChIP-seq (Fig. S12D). This includes *GAT204* and *LIV3*, encoding two transcription factors implicated in virulence whose direct target genes overlap with those of Gat201 (27), and the barwin-like protein gene, *BLP1*, that is required for the antiphagocytic function of Gat201 (23). Additionally, a small number of transcripts were differentially expressed depending on serum (Fig. S11), consistent with an overall modest impact of serum on *GAT201*-dependent growth.

**Fig 2:**
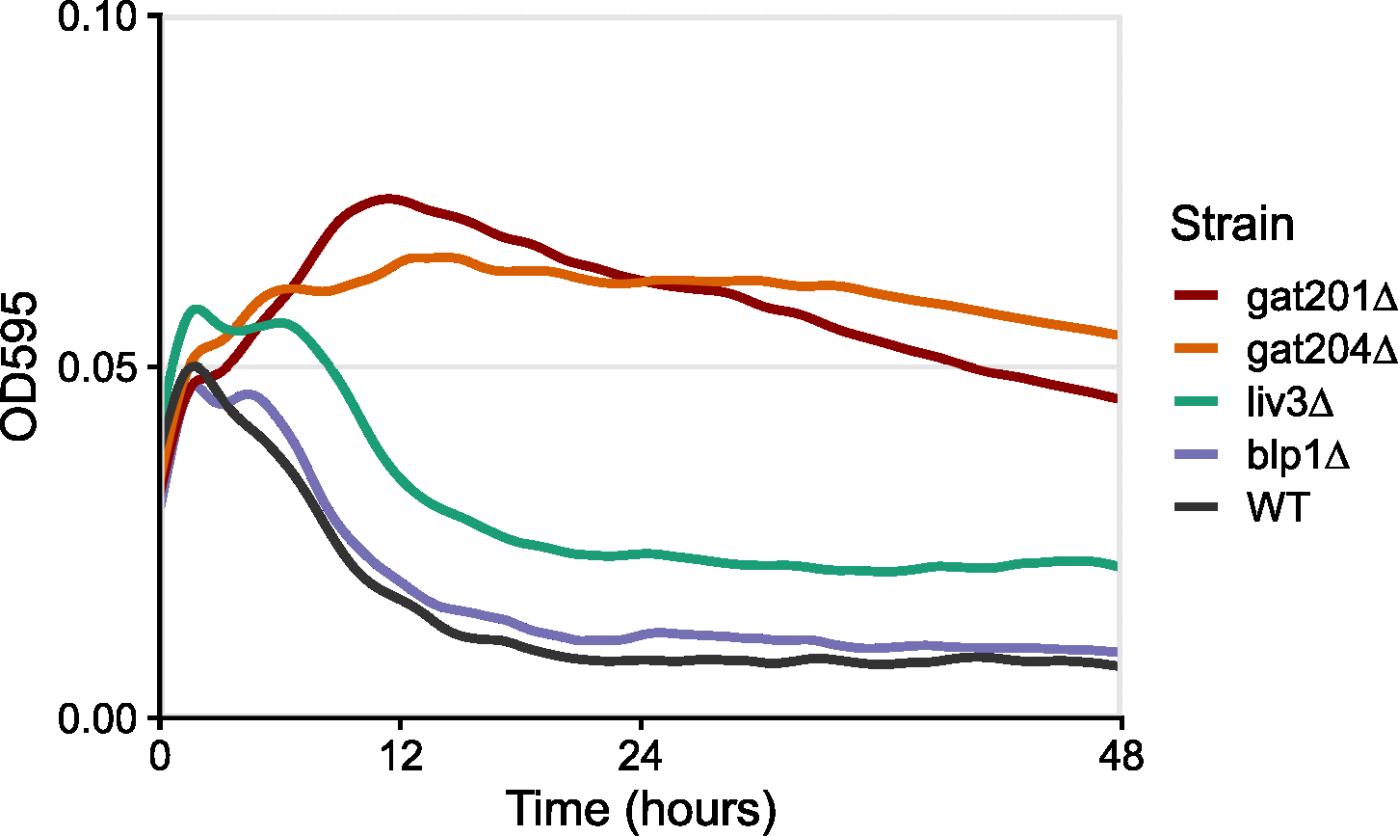
*GAT201* co-factors *GAT204* and *LIV3* also restrict growth. Growth curves of optical density at 595 nm (OD_595_) were collected via plate reader from four biological replicates, three technical replicates each, at 37°C. Fig. S13 shows individual replicates.

To test which Gat201-regulated genes might contribute to restricted growth in alkaline media, we performed growth analysis on a selection of deletion mutants. In RPMI medium, *gat204*∆ behaves similarly to *gat201*∆ by increasing in density, unlike wild-type cells that decline in density within 10 h (Fig. 2; Fig. S13). An intermediate density is shown by *liv3*∆ (Fig. 2; Fig. S13), whereas the double mutant *gat204*∆ *liv3*∆ grows similarly to *gat204*∆*,* growing better than wild-type in RPMI but not as well as *gat201*∆ (Fig. S14). Conversely, *BLP1* is dispensable for the growth phenotype: *blp1*∆ cells grow similarly to wild-type cells in RPMI (Fig. 2; Fig. S13). Deletion of other *GAT201* targets that we tested did not relieve restriction of growth, including transcription factors *PDR802* and *ECM2201*, and metalloproteinase *MEP1* (Fig. S13). Overall, these data show that a *GAT201*/*GAT204*/*LIV3*-dependent pathway restricts growth in alkaline conditions.

### *GAT201* represses growth at alkaline pH but is required for growth in RPMI at neutral pH

We next examined the role of pH in determining the restricted growth phenotype. Our initial experiments were conducted in an RPMI formulation buffered with sodium bicarbonate and grown under aerobic conditions, beginning at neutral pH and rising to alkaline pH over the initial hours of the experiment (30). This means that the 2-h time point reactivation and transcriptional phenotypes reflect growth between pH 7.0 and pH 8.5, but the longer-term decline in growth and viability occurs at pH approximately 9.5. We observed that in an alternative RPMI-like “CO_2_-independent media,” which maintains near neutral pH with a phosphate-based buffering agent, both *GAT201* and *gat201*∆ cells continue to increase in density over a 72-h period (Fig. S15). This differential growth in media with otherwise identical nutrient composition suggested that the buffering agent and/or pH was responsible for the phenotype.

**Fig 3:**
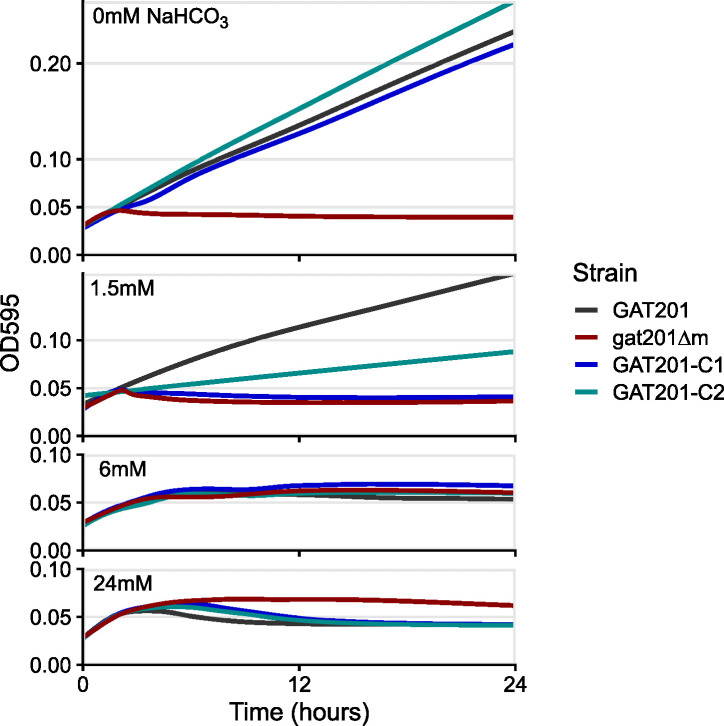
The effect of *GAT201* on growth depends on sodium bicarbonate (NaHCO_3_). Starting with an RPMI formulation lacking NaHCO_3_, we added either 0 mM, 1.5 mM, 6 mM, or 24 mM NaHCO_3_ and grew *Cryptococcus* for 24 h. Wild-type *GAT201* cells grow in 0 mM NaHCO_3_ but do not grow in 24 mM NaHCO_3_, whereas *gat201*∆ cells have opposite phenotypes of no growth in 0 mM and growth at 24 mM. These cells have intermediate phenotypes at intermediate concentrations of NaHCO_3_, whereas complemented strains have growth phenotypes resembling wild-type. This figure shows the median of three technical replicates from a single biological replicate, and two further biological replicates are shown in Fig. S16.

To isolate the effect of sodium bicarbonate (NaHCO_3_) on growth, we grew cells in unbuffered RPMI base with varying concentrations of NaHCO_3_ and under aerobic conditions. With 24 mM NaHCO_3_, the same as in the standard RPMI formulation used previously, we again observed that wild-type *GAT201* cells do not grow, but *gat201*∆ cells do grow, in long-term cultures (Fig. 3; Fig. S16). Surprisingly, at lower concentrations of NaHCO_3_, this effect changes, and in RPMI with no added NaHCO_3_ (neutral pH), wild-type cells grow consistently, whereas *gat201*∆ cells do not grow (Fig. 3; Fig. S16). Complementing *GAT201* into deletion strains restored the wild-type growth phenotype of growth at 0 mM NaHCO3 and arrest at 24 mM NaHCO_3_.

To interpret these data, note that bicarbonate ions in the media are in exchange with carbon dioxide in the air around the cultures (30). Addition of 24 mM NaHCO_3_ leads to an equilibrium pH of about 7.5 in 5% CO_2_ (30); however, at atmospheric CO_2_ (roughly 0.04%), this medium reaches pH ~9.5 within hours, depending on culture volume and shaking. Without any added NaHCO_3_, the media pH remains close to neutral. Thus, the major differences in growth phenotypes that we observe occur after equilibration to a more alkaline pH. Further work will be needed to dissect the effect of pH, buffers, and exogenous CO_2_ on *GAT201*-dependent growth, but the phenotype that *GAT201* promotes growth in RPMI media at near-neutral pH and represses growth at alkaline pH is reproducible in our hands.

Because cyclic AMP-dependent signaling through the Rim101 transcription factor also regulates growth at alkaline pH (31, 32), we tested whether cyclic AMP signaling affects our observed *GAT201*-dependent phenotype. We found that the addition of exogenous cAMP does not substantially affect growth in RPMI with or without NaHCO_3_ added: again, wild-type *GAT201* cells grow far more than *gat201*∆*m* in the absence of NaHCO_3_, and the phenotype is reversed in 24 mM NaHCO_3_ (Fig. S17). This shows that the GAT201 pathway is largely independent of cAMP signaling, and thus of Rim101 signaling, indicating a distinct alkaline-responsive pathway controlled by *GAT201*.

### *C. neoformans* Gat201 is homologous to other GATA-family zinc finger proteins that regulate fungal growth and environmental responses

**Fig 4:**
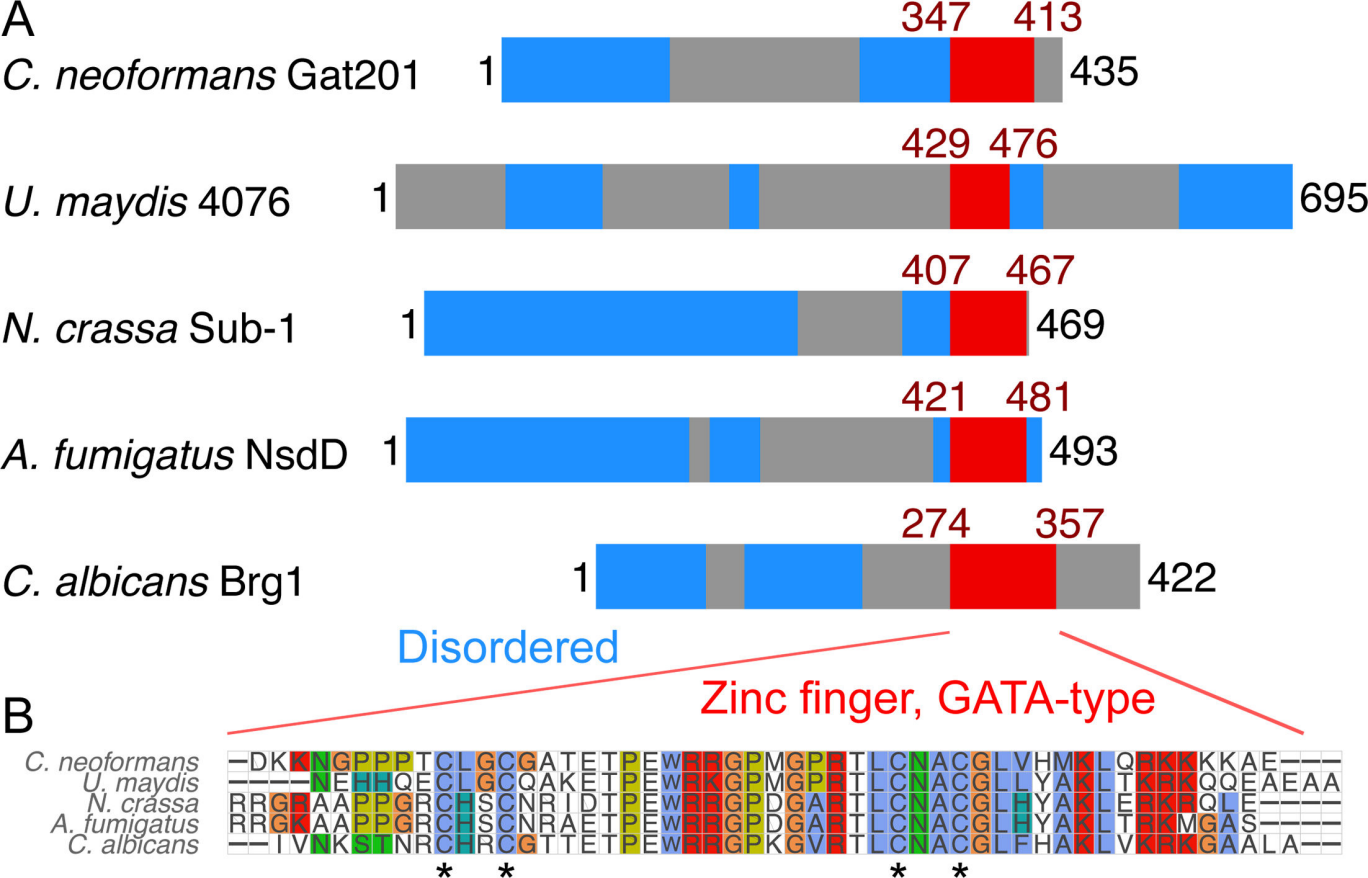
*C. neoformans* Gat201 is homologous to other GATA-family zinc finger proteins that regulate fungal growth and environmental responses. (A) Domain structure of Gat201 and 4 close homologs, with GATA-like zinc finger domain shown in red (Interpro IPR013088) and predicted unstructured regions in blue (MobiDB Lite consensus disorder), taken from Interpro (35). (B) Multiple sequence alignment of the GATA-like zinc finger domains of homologs made with MUSCLE (36). Conserved cysteine residues typical of GATA-like zinc fingers are indicated with asterisks. An extended phylogeny and homology analysis are shown in Fig. S18.

We asked if Gat201 could be homologous to other fungal transcription factors that might indicate a conserved pathway. Gat201 is a 435 amino acid-long protein predicted to have only a single structured domain of 58 amino acids near the C-terminus, the GATA-like zinc finger domain (Fig. 4A). This domain is found across a broad variety of transcription factors that integrate environmental signals and metabolism (33). Searching for homologs of *C. neoformans* Gat201 by BLASTP (34) detects many proteins with GATA-like domains and a variety of domain structures, consistent with the known diversity in GATA-like transcription factors (33). Because there are multiple GATA-like domain proteins in each fungal species that we examined, we turned to more precise analyses to search for true homologs of Gat201 (see Materials and Methods).

Our analysis grouped *C. neoformans* Gat201 with a subset of other proteins containing a single C-terminal GATA-like domain: *Ustilago maydis* UMAG_04076, *Neurospora crassa* Sub-1, *Aspergillus fumigatus* NsdD, and *Candida albicans* Brg1 (Fig. 4; Fig. S18). Of these, synteny analysis (37) showed that only *U. maydis* UMAG_04076 is in a conserved gene order with *GAT201* (see supplemental results). Several of these homologs have reported roles in regulating growth and environmental responses (38–41). The GATA-like zinc-finger domains of these Gat201 homologs are highly conserved (Fig. 4B; Fig. S18B), including the four cysteines that coordinate the zinc ion (33). In addition, the AlphaFold2 protein structure database predicts a short unannotated alpha-helix-rich domain in *C. neoformans* Gat201, *N. crassa* Sub-1, and *A. fumigatus* NsdD (42).

Within a larger set of transcription factors with a single GATA domain (Fig. S18), fungal Gat201-like transcription factors are most closely related to amoeba homologs, including gtaI, a GATA-family transcription factor that is required for morphological transitions in *Dictyostelium discoideum* (43). *S. cerevisiae* Gat2, Gat3, and Gat4 were grouped in a separate clade, whereas *C. neoformans* Gat204 was grouped into another clade with a different set of amoebal homologs (Fig. S18). The phylogenetic identification of a Gat201-like subfamily is further supported by bootstrap analysis, by the similarity of the GATA domains, and by synteny analysis (37) (Fig. S18 and supplemental results). Future work will be needed to assess which of Gat201’s predicted homologs have conserved molecular function or operate in a conserved pathway.

## DISCUSSION

### Gat201 is part of an alkaline-restricted growth pathway

Proliferation is a major driver of *C. neoformans* pathogenesis: cryptococcosis pathology is driven by the accumulation of yeast in diverse host niches, and high fungal burden is a strong correlate of poor outcomes (11, 44–46). To proliferate in the host and cause disease, *C. neoformans* yeast must rapidly adapt to the lung environment, characterized by nutrient limitation, high temperature (37°C), CO2, and, within airways, transiently high pH (>8.5) (21). In this study, we modeled the early events of the fungal transition from stationary phase to growth in cell culture media at moderate to high pH and found that mRNA encoding the virulence-associated transcription factor *GAT201* plays a central role. Surprisingly, *GAT201* was associated with restriction in proliferation—loss of budding, growth, and, later, viability—but only at high pH.

**Fig 5:**
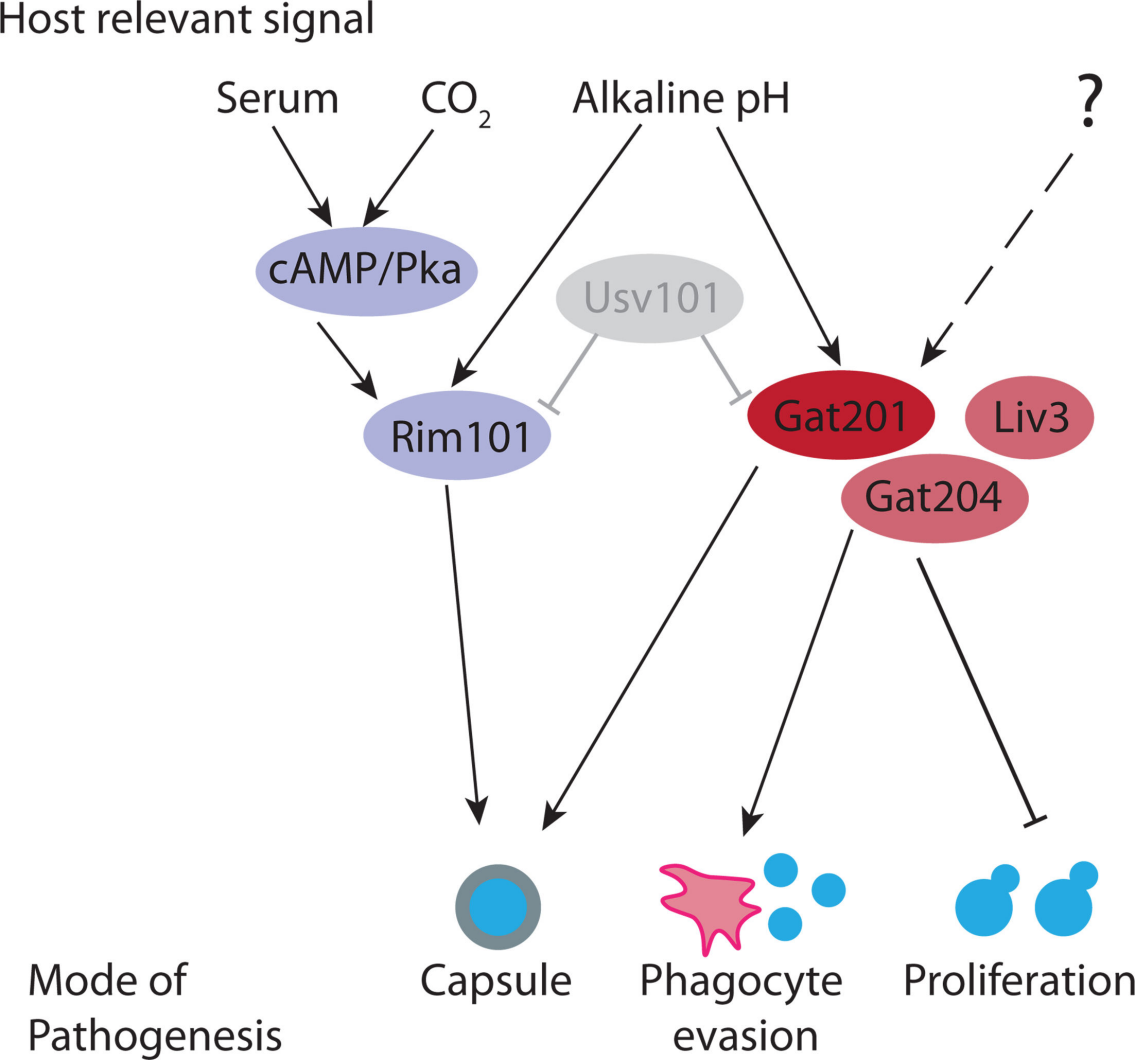
The Gat201 pathway promotes *Cryptococcus* virulence and represses proliferation. Gat201 acts in parallel to the serum-responsive cAMP/Pka pathway and the major pH-responsive Rim101 pathway. Gat201 requires mutual activators, Gat204 and Liv3, to suppress proliferation.

These data suggest that Gat201 is part of an alkaline-restricted growth (ARG) pathway that responds to environmental signals, including alkaline pH, to restrict cell proliferation and promote the synthesis of defensive capsule (Fig. 5). Our observation that deletion of *GAT201* restores yeast cell budding, in addition to disrupting the production of capsule, is consistent with previously published microscopy data showing both reduced capsule production and also increased budding that was not the focus of that study (25). Also consistent, we measured a 10-fold increase in *GAT201* mRNA abundance within 30 min of inoculation in RPMI media, suggesting the existence of fast-acting upstream pathway components that induce *GAT201* transcription and/or stabilize the *GAT201* transcript. Downstream, Gat201 regulates hundreds of targets, including other transcription factors that are implicated in *Cryptococcus* virulence, as well as many poorly characterized genes. We observed upregulation of many direct targets, that is, genes whose promoters are bound by Gat201, including transcriptional co-factors *GAT204* and *LIV3,* which we also found are required for alkaline-restricted growth. Together, these findings point to Gat201-Gat204-Liv3 as a core regulatory modulator of proliferation and viability (Fig. 5). Functional assays for Gat201 pathway activation will enable dissection of the pathway and its involvement in proliferation and could shed light on Gat201’s role in promoting virulence. Future work could map pathway components using forward genetic approaches that exploit growth conditions where the functional Gat201 pathway renders *Cryptococcus* inviable.

### The Gat201 pathway is independent of previously characterized alkaline-responsive pathways

*C. neoformans* is extremely sensitive to alkaline pH, failing to grow above pH 8.5, a limitation unusual among fungal pathogens (47, 48). In contrast to *C. neoformans*, ascomycete pathogens are more alkaline tolerant above pH 8.5: *Aspergillus* species are tolerant to pH 11 (49), and *Candida* species can tolerate alkaline conditions ranging from pH 10, for *C. albicans*, to pH 13 for *C. auris* and *C. parapsilosis* (50). In ascomycetes and basidiomycetes, alkaline growth is enabled by the transcription factor Rim101/PacC (51). Previous studies in *C. neoformans* have focused on genes whose loss further restricts growth at alkaline pH, including *RIM101* (52). The GAT201 pathway appears to be independent of the Rim101 pathway: *RIM101* transcript expression does not depend on *GAT201* (Fig. S9), nor does *GAT201* expression depend on *RIM101* (53), and our comparison of Gat201-dependent and Rim101-dependent transcriptional profiles revealed no statistical enrichment for shared targets (data not shown). Rim101 acts downstream of the cAMP pathway, and exogenous cAMP does not change the *GAT201*-dependent growth phenotypes (Fig. S17). Also, the expression of other genes necessary for growth in alkaline conditions, such as *PHO4* (54), *ENA1* (55)*, ECA1* (56), *CAN2*, or *CAC1* (57), or the sterol homeostasis pathway regulated by the transcription factor Sre1 (58), was not dependent on *GAT201*. This argues that the Gat201 pathway regulates growth in alkaline conditions independently of previously characterized pathways.

### Gat201 is a member of a conserved family of environmentally responsive transcription factors

Our analysis of Gat201 homologous proteins in basidiomycetes and ascomycetes (Fig. 4) also suggests hypotheses for future investigation. We found a syntenic homolog of *GAT201* in the basidiomycete plant pathogen *Ustilago maydis*, UMAG_04076. Interestingly, *U. maydis* also exhibits alkaline-restricted growth that is Rim101-independent, but the causative pathway is unknown (59). The predicted *Neurospora* homologous protein, Sub-1, co-regulates genes downstream of the light-responsive white collar complex, connecting light responses and fungal development (60). The *Aspergillus* homologous protein, NsdD, is a crucial regulator of sexual development (61, 62). Orthologous Brg1 in *Candida albicans* is required for hyphal growth, biofilm formation, and virulence (63). Collectively, this suggests that Gat201 may be part of a conserved family of GATA transcription factors that regulate proliferation and morphology in response to environmental stimuli. Defining the regulatory targets, co-factors, and upstream signaling pathways leading to Gat201 family activation in different species would reveal the degree of functional conservation.

### How does *GAT201* restrict growth?

The *GAT201*-dependent transcriptional profiles during alkaline-restricted growth provide some insight into the mechanisms of growth restriction. *GAT201*-dependent downregulation of ribosome biogenesis genes indicates that protein synthesis, a core pathway required for growth, is repressed downstream of *GAT201* during reactivation in RPMI. By contrast, we observed ribosomal proteins to be strongly induced in wild-type cells reactivating in rich YPD media. This shows that *Cryptococcus* grown in alkaline RPMI represses biosynthetic and replicative processes while also upregulating capsule production. It has previously been shown that capsule synthesis is restricted to the G1 phase of the cell cycle, while budding occurs in G2 (16), consistent with our observation that populations with reduced capsule have more buds within the same time period.

We propose three non-exclusive hypotheses for how Gat201 restricts growth. First, the Gat201 pathway could restrict growth through transcriptional regulation of a core cell cycle pathway. Second, the Gat201 pathway could promote capsule production, and the redirection of biosynthetic resources to capsule production could result in restricted growth. Third, some factors required for nutrient acquisition could be regulated downstream of Gat201, and a failure to acquire some essential nutrients would restrict growth. Although nutrient acquisition could be a key contributor, we can exclude that nutrient content alone explains growth restriction because *GAT201* and *gat201*∆ cells behave differently in identical nutrient-rich RPMI media. Furthermore, modulation of media pH and buffering agents alone, without changing nutrient composition *per se*, is sufficient to alter *GAT201*-dependent growth phenotypes. Alkaline conditions reduce the availability of H+ ions, which are important for the transport of nutrients across the cell membrane. Although we observed no *GAT201* dependence for the expression of known H+ pumps required for alkaline growth, several transmembrane transporters are upregulated in *GAT201* cells compared with *gat201*∆. Another recent report found that deletion of *GAT201* or *GAT204* also promotes growth in near-neutral buffered media with 5% CO_2_ (64), which could be related to the phenotype that we report here. Future work will need to investigate these mechanisms.

## MATERIALS AND METHODS

### Strains

Wild-type *C. neoformans* H99 (65), KN99a, and KN99alpha (29) were gifts from Andrew Alspaugh, Duke University, NC, USA. Wild-type Gat201 mutants *gat201*∆*m* from the Madhani lab *C. neoformans* deletion collection (22), and *gat201*∆*b* from the Bahn lab transcription factor disruption collection (24) were obtained from the Fungal Genetics Stock Center (Manhattan, KS, USA; https://fgsc.net). We verified the gene disruption/deletions by PCR and Sanger sequencing. Other deletion mutants used in this paper are also from the Madhani lab *C. neoformans* deletion collection (22).

To complement *gat201*∆, the GAT201 gene, including native promoter, terminator, and introns with a HYG selection marker (pGAT201-cGAT201-tGAT201-HYG) was integrated into a genomic safe haven locus 4 on chromosome 7 (66) in the *gat201*∆*m* strain. Two independent clones were taken from the transformation reaction and verified by PCR, *GAT201-C1* and *GAT201-C2*. We made the integration constructs using modular cloning by Möbius assembly (67), the details of which we will explain in another publication. A full plasmid map is included in Table S4. We integrated this construct using a *Cryptococcus* CRISPR-Cas9 system (68).

### RNA-seq experiments

Full details are in the supplemental materials and methods.

For RNA-seq data set 1 (reactivation), wild-type *C. neoformans* H99 were grown in liquid YPD for 5 days to make “stationary phase” cells. Aliquots of stationary phase culture were pelleted and resuspended in pre-warmed media conditions: 25°C YPD, 37°C YPD, 25°C RPMI 1640 + 10% heat-inactivated fetal calf serum (HI-FCS), and 37°C RPMI+ 10% HI-FCS, 100 mL each, and incubated at 60 rpm. Two biological replicates were collected on successive days.

For RNA-seq data set 2 (GAT201 dependence), strains KN99a and KN99alpha, *gat201*∆*m*, and *gat201*∆*b*, were grown for 5 days in liquid YPDA. Aliquots of these stationary phase cultures were inoculated in fresh pre-warmed media, RPMI or RPMI+ 10% HI-FBS, and incubated at 37°C with 60  rpm shaking. Samples were collected at 30 min, 2 h, and 4 h. Two biological replicates were performed for each of the four strains.

For RNA extraction, *C. neoformans* cultures were fixed in methanol and dry ice, lyophilized, lysed by bead-beating in denaturing RLT buffer or TRI reagent, and RNA extracted using the Qiagen RNeasy Plant Mini Kit (Qiagen, Valencia, CA, USA). RNA-seq data set 1 (reactivation) libraries were prepared with the RNATag-Seq protocol with rRNA depletion (69), and data set 2 (*GAT201* dependence) was prepared with QuantSeq FWD 3’ mRNA-Seq Library Prep Kit (Lexogen, Vienna, Austria). Full details are in the supplemental materials and methods.

### RNA-seq bioinformatic and statistical data analyses

Complete analysis code for the RNA-seq data sets from raw reads onwards is found at https://github.com/ewallace/CryptoWakeupRNASeq and doi:10.5281/zenodo.11506094 (data set 1) and at https://github.com/ewallace/CryptoGat201RNASeq and doi:10.5281/zenodo.12207483 (data set 2).

In summary, basic assessments of sequence data quality were performed using FastQC (70) and MultiQC (71). Raw sequencing reads were trimmed and filtered using Cutadapt (72). Sequenced reads were aligned to the *C. neoformans* H99 reference sequence CNA3 (73) using HISAT2 (74). We used featureCounts (75) to assign mapped reads per gene using the longest transcript per gene annotation from (76). Gene expression was normalized using the regularized logarithm (rlog) function from DESeq2 (77). We evaluated the gene expression differences using a test based on a negative binomial distribution, also in DESeq2 (77), using a 5% false discovery rate calculated by the “p.adjust” function in R using the Benjamini and Hochberg method (78).

Gene clustering was performed by using (1 − correlation) as a distance metric, then hierarchical clustering in UPGMA using R’s hclust function (79). After a lengthy iterative process in which many methods were evaluated, representative clusters of genes were selected from the hierarchical clusters using R’s cutree function with user-defined numbers of groups (k option). Full details and analysis code are in the repositories.

Differentially expressed genes and gene clusters were subjected to GO term enrichment analysis using the online resources at FungiDB (80).

### Microscopy

For the stationary phase, cells were revived from −80°C glycerol stocks on YPD agar, and within 2 days, single colonies were inoculated into 10 mL liquid YPD (1% yeast extract, 2% Bactopeptone, 2% Dextrose) and incubated for 5 days at 200 rpm, 30°C. On day 5, the temperature was reduced to 25°C, and the cells were allowed to adjust for >4 h. Cells were counted using a hemocytometer, and 1 mL of the pellet (10^6^ cells) was collected, washed 1× with PBS, split into four tubes, and then resuspended in the appropriate pre-warmed medium as indicated to a final volume of 10 mL each. Cells were incubated in the indicated condition for 120 min, and then the entire pellet was collected and fixed with 4% methanol-free formaldehyde (Pierce) for 10 min, then washed 3× with PBS. India ink (Remel) slides were prepared, and cells were imaged using an inverted Zeiss AxioObserver Z1 with a Plan-Neofluor 40×/1.3 numerical aperture (NA) oil immersion lens objective (Carl Zeiss) and a 16-bit CoolSNAP H2 charge-coupled-device (CCD) camera (Photometrics). For each figure, three biological replicates were initiated using independent stationary cultures and collected serially on the same day. The entire experiment was performed independently twice.

### Growth curves

Cells from a single colony were inoculated into 5 mL liquid YPDA (1% yeast extract, 2% Bacto-peptone, 2% dextrose, 0.002% adenine) and incubated for 20 h at 180 rpm, 30°C. Cells were washed with ddH_2_O and resuspended in the required volume of the appropriate media to an initial OD at 595 nm of 0.2. Wells in the microplate were filled with this suspension (200 µL in each well). The absorbance in each well was measured at 595 nm at given intervals (10 min) with shaking (300 rpm for 1 min) directly prior to reading. Reference measurements were performed on the outer wells where 200 µL of media only was added. The microplate was incubated in the Tecan Infinite® 200 PRO plate reader at 37°C for 48 or 72 h. Cells were grown as stated in RPMI 1640 (Sigma R8758) with or without heat-inactivated serum (Sigma F9665), or YPDA, for figures except where noted. For Fig. S15, we used CO_2_-independent media, buffered with mono and dibasic sodium phosphate and β-glycerophosphate (Gibco/ThermoFisher 18045088). For Fig. 4; Fig. S16 and S17, we used RPMI media without phenol red and without NaHCO_3_ (Sigma R8755), adding dibutyryl cAMP (Sigma D0627) or NaHCO_3_ from aqueous stock solutions to the indicated final concentration. For these, we were particularly careful to rapidly prepare media and then inoculate cells for growth curves, for a reproducible pH of the growth media.

### Colony-forming unit assay

Cells from a single colony were inoculated into 25 mL liquid YPDA (1% yeast extract, 2% Bacto-peptone, 2% dextrose, 0.002% adenine) and incubated for 5 days at 150 rpm, 30°C. Cells were washed with ddH_2_O and resuspended in a total volume of 20 mL RPMI at an OD 595 nm of 0.1 for each strain. Cultures were incubated at 37°C, 60 rpm and samples were collected at 12 h intervals (0–60 h). Serial dilutions were prepared for each sample collected from each strain down to 10^−4^; 100 μL of dilutions 10^−3^ and 10^−4^ were plated onto YPDA agar plates and incubated at 30°C for 48 h. Plates were imaged using an ImageQuant 800 (Amersham/Cytiva, settings: Colorimetric, OD measurement, Auto exposure, Capture area = 160 × 220 nm), and the resulting colonies were counted. Three biological replicates were collected for each strain.

### RT-qPCR

Cells from a single colony were inoculated into 5 mL liquid YPDA (1% yeast extract, 2% Bacto-peptone, 2% dextrose, 0.002% adenine) and incubated for 20 h at 180 rpm, 30°C. Cells were washed with ddH_2_O and resuspended in 100 mL RPMI (Sigma R8758) at an initial OD ar 595 nm of 0.1, incubated at 37°C, 150 rpm for 7 h. Cells were fixed in methanol, and RNA was extracted using mechanical disruption in TRIzol with zirconium beads, followed by the Qiagen Plant and Fungal Extraction Kit; 100 ng of purified RNA was used from each sample to synthesize cDNA using Superscript IV Reverse Transcriptase (Invitrogen) and random primers (NEB). Samples were DNase treated prior to reverse transcription. QPCR was carried out using Brilliant III Ultra-fast SYBR Green qPCR mix (Agilent) with appropriate target gene primers. mRNA expression of GAT201 (Forward primer: 5′-ACCACGAGTCTTGGGATAGA-3′, Reverse primer: 5′-CTGGGTGTTCGGGATAAAGTAG-3′), GAT204 (Forward primer: 5′-CCACCTCTTCCTTCCTTGTTAAA-3′, Reverse primer: 5′-GTCTGCCATCGTCGTACTAATG-3′), and LIV3 (Forward primer: 5′-CCTCTTCCACTTCCACATCAA-3′, Reverse primer: 5′-GGTCTCGGCACAGCATATT-3′). Test genes were compared with three reference genes ACT1 (Forward primer: 5′-GTGGTTCTATCCTTGCCTCTTT-3′, Reverse primer: 5′-CACTTTCGGTGGACGATTGA-3′), GPD1 (Forward primer: 5′-TCGAGCAACGTCTTGGTATC-3′, Reverse primer: 5′-GCTCTCCATCCTCCTTGTTT-3′), and SRP14 (Forward primer: 5’--3’, Reverse primer: 5’--3’). The data were analyzed with tidyqpcr (81).

### Analysis of Gat201 homology

We selected the proteins closest to *C. deneoformans* Gat201 in the PANTHERDB curated homology database within the family GATA transcription factor, PTHR45658 (82). This family groups Gat201 both with fungal and amoebal proteins that have the same domain structure, including *S. cerevisiae* Gat2 (YMR136W) and *C. neoformans* Gat204, and also with wc-2 transcription factors that are involved in light responses in filamentous fungi and that have an additional PAS sensing domain (83). We filtered to only homologs with the same domain structure as Gat201, that is, no PAS domain, and performed a full-length protein alignment seeded with the GATA domain using MAFFT (84) and calculated a phylogenetic tree by Bayesian maximum likelihood using IQ-TREE (85). The complete list of proteins is presented in https://github.com/ewallace/Gat201homology_2022/.

### Data analysis

Data analysis scripts and raw data for budding index assay, growth curve assay, CFU assay, and RT-qPCR are in the repository https://github.com/ewallace/CryptoGat201_2023_suppdata and doi:10.5281/zenodo.11506135. Scripts and raw data for the homology analysis are in the repository https://github.com/ewallace/Gat201homology_2022/ and doi:10.5281/zenodo.11506156. Data were analyzed in the statistical open-source language R (86), making extensive use of the tidyverse for data manipulation (87) and ggplot2 for figures (88). Additional figures were prepared in Inkscape (The Inkscape Team, https://inkscape.org/).

## Data Availability

RNA-sequencing data are available on Gene Expression Omnibus (GEO) under accession numbers GSE133067 (data set 1) and GSE217345 (data set 2).

## SUPPLEMENTAL MATERIAL

Supplemental material msphere.00191-25-s0001.docx Supplemental results, Fig. S1-S18, supplemental materials and methods, and captions for supplemental tables.

Table S1 msphere.00191-25-s0002.xlsx RNASeq data set 1 gene clusters.

Table S2 msphere.00191-25-s0003.xlsx RNAseq data set 1 DGE analysis.

Table S3 msphere.00191-25-s0004.xlsx RNAseq data set 2 DGE analysis.

Table S4 msphere.00191-25-s0005.txt Plasmid map for GAT201 complementation construct, in GenBank format.

ASM does not own the copyrights to Supplemental Material that may be linked to, or accessed through, an article. The authors have granted ASM a non-exclusive, world-wide license to publish the Supplemental Material files. Please contact the corresponding author directly for reuse.
