# Supplementary material for: The GATA-like transcription factor Gat201 determines alkaline-restricted growth in Cryptococcus neoformans
Source: mSphere. 2025 Jun 4;10(6):e00191-25. doi: 10.1128/msphere.00191-25 (PMC12188746; doi:10.1128/msphere.00191-25)
Supplement: Supplemental material — Supplemental results, Fig. S1-S18, supplemental materials and methods, and captions for supplemental tables. [file msphere.00191-25-s0001.docx]

**Supplementary material for:**

**The GATA-like transcription factor Gat201 determines alkaline restricted growth in *Cryptococcus neoformans***

Elizabeth S. Hughes^1^, Laura R. Tuck^1^, Zhenzhen He^1^, Elizabeth R. Ballou^2^, Edward W.J. Wallace^1^.

1. Institute for Cell Biology, and Centre for Engineering Biology, School of Biological Sciences, The University of Edinburgh. 2. MRC Centre for Medical Mycology, The University of Exeter.

**List of Supplementary tables:**

**Table S1. RNASeq dataset 1 Gene Clusters.**

**Table S2. RNAseq dataset 1 DGE analysis.**

**Table S3. RNAseq dataset 2 DGE analysis.**

**Table S4. Plasmid map for GAT201 complementation construct.**

**Supplementary Results, including Figs S1-S18.**

**RNA-seq timecourse of C. neoformans reactivation.**

To investigate the early events of cellular reactivation, we aimed to identify meaningful trends in gene expression and cell morphology across a time-course of reactivation in different growth conditions. We inoculated stationary phase cells incubated for 5 days in YPD at 30°C into four growth conditions (YPD or RPMI at 25°C or 37°C). We sampled cells prior to inoculation (0 minutes), and then sampled the cultures at 10, 30, 60, and 120 minutes following inoculation. We also collected a standard growth condition, mid-exponential phase cells reinoculated 1:30 into fresh YPD at 30°C for 180 min. We chose to sample 4 timepoints per condition in 2 biological replicates each, which is sufficient for statistical analysis and differential gene expression using the DESeq2 package [(77)](https://paperpile.com/c/bq2XYM/PftD). Cells were examined for morphology (Fig S1B) and for transcriptional changes via RNA-seq (Fig S1C, D, E)

**Fig S1 (next page): *Cryptococcus neoformans* rapidly induces media-specific growth programs upon reactivation from stationary phase.** A, Design of time-course experiment to measure the contribution of media and temperature on reactivation of stationary phase cells. B, Budding (yellow-green arrow) is seen in YPD at both temperatures (top row) while capsule (light red arrow) is primarily seen in RPMI + serum at 37°C (bottom right). Micrograph shows India Ink staining of cells taken from one replicate of cells used for the RNA-seq experiment, 150 minutes after inoculation. C, Time from inoculation and media dominate the overall variance in gene expression, shown by principal component analysis on the regularized log-counts per gene in every replicate. Each replicate is plotted and labeled by the timepoint, with colours as in Fig S1A (grey - 0 min, yellow - YPD 25°C, orange - YPD 37°C, light purple - RPMI 25°C, dark purple - RPMI 37°C), additionally with dark green for exponential phase rich media 30°C. D, Distinct clusters of co-regulated genes respond to reactivation in different media, shown by clustered heatmap of log2 fold-change per gene calculated by DESeq2. Each row represents an individual gene and rows are clustered by co-expression patterns (see methods), while each column represents the regularized log2 fold-change estimate across both replicates in a single condition. E, Representative genes from different clusters show distinct expression patterns, again in regularized log2 fold-change per gene. Colours of growth conditions are as in Fig S1A.


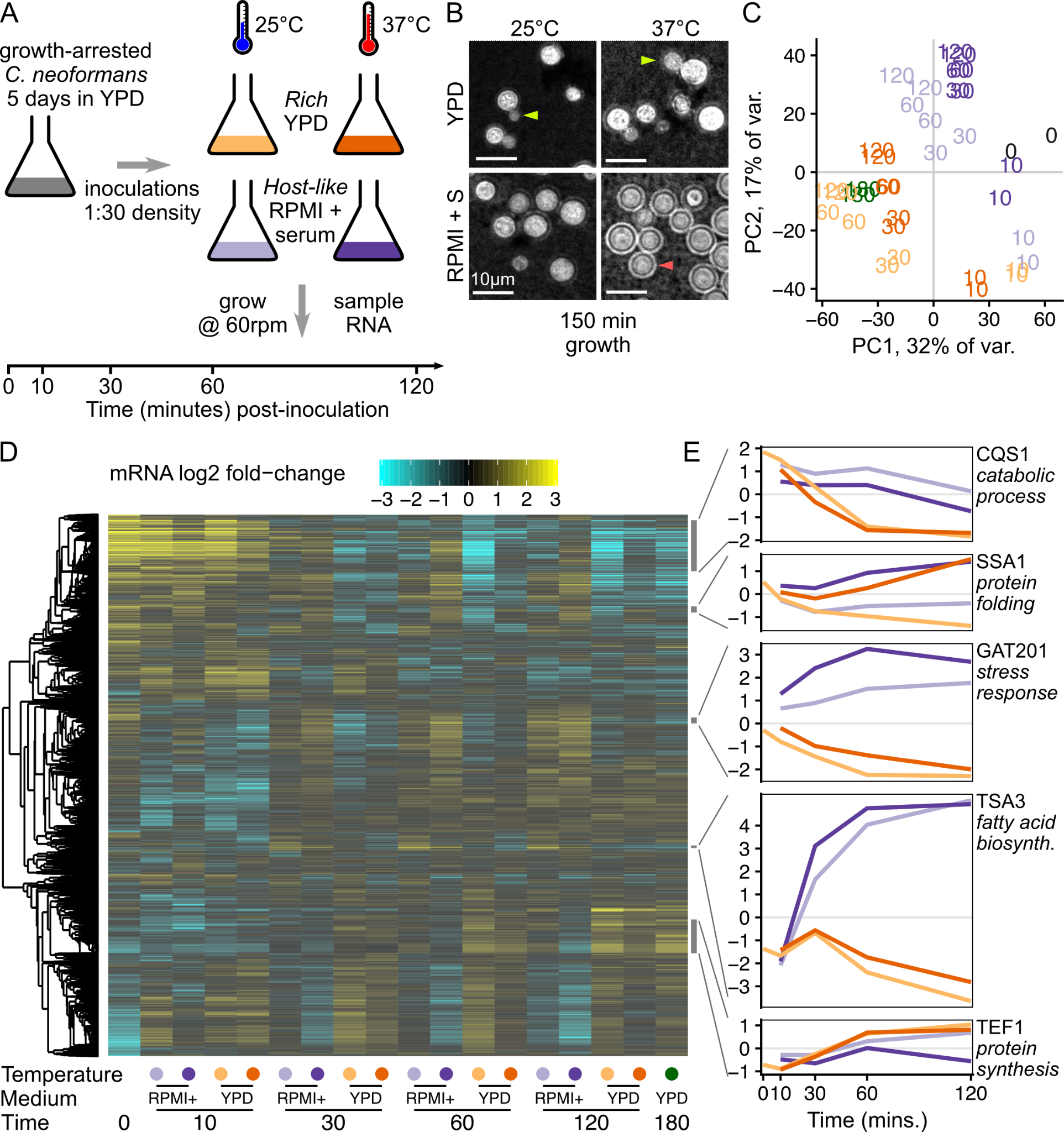


We observed that cells reactivating in rich medium resumed growth and cell division, with visible buds 150 minutes after inoculation (Fig S1B). However, cells reactivating in RPMI medium showed no visible budding, and when grown at 37°C also produced a polysaccharide capsule visible with India Ink staining (Fig S1B). The RPMI media here is buffered with sodium bicarbonate, grown in a standard aerobic shaking incubator at 60 rpm.

Consistent with observed changes in cell morphology, we identified distinct gene expression programs in the different media and temperatures that were consistent across replicates and timepoints. Principal component analysis on the regularized log-counts per gene shows clear separation between growth conditions (Fig S1C and Fig S2). Principal components (PCs) 1 and 2 separate stationary phase cells and early timepoints (0 min, 10 mins) from later timepoints. PCs 1 and 2 also separate YPD media and RPMI media at later timepoints, while the exponential phase samples grown in rich media at 30°C group with the other rich media samples. PC3 separates samples grown at different temperatures with a clear divide between 25°C and 37°C (Fig S2). PC4 largely separates the 0 min samples from all later timepoints (Fig S2), emphasizing that induction of gene expression pathways is detectable after only 10 minutes in either growth medium, and independent of temperature. PC analysis groups both biological replicates together for all sample conditions, indicating a reproducible experiment.

Distinct transcriptional pathways are activated during cellular reactivation (Fig S1D, S1E, S3). Consistent signatures include: known stationary phase genes are downregulated in YPD (e.g. *CQS1*, encoding Cryptococcus quorum sensing protein Qsp1); known heat-shock proteins are relatively upregulated at 37°C in both media (e.g. SSA1, encoding an HSP70); protein synthesis genes are upregulated in YPD and less so in RPMI (e.g. *TEF1*, encoding translation elongation factor 1α). Two distinct clusters of genes are upregulated in RPMI compared to YPD: one including the virulence-associated transcription factor *GAT201* and enriched for stress response genes, and another including thiol-specific antioxidant protein *TSA3* and enriched for fatty acid biosynthesis genes. Analysis with DESeq2 showed these to be significantly differentially expressed, for example *GAT201* is one of the most differentially expressed genes after 1 hour at both 37°C (~24-fold, p < 10^-19^) and 25°C (~13-fold, p < 10^-20^). Consistent with *GAT201* induction from 10 minutes after inoculation, we observed induction of Gat201 targets, including *GAT204* and *LIV3* expression, in RPMI at 30 minutes and onwards (Fig S3). The complete clustering analysis and differential gene analysis code along with gene lists is found in the accompanying code repository.

Clustering genes by their expression patterns reveals the combinatorial impact of media and temperature over time and given the high reproducibility of biological replicates we summarise with a regularized log-mean value for each condition, calculated by DESeq2 (Fig S1D). One cluster of 484 genes are highly expressed in stationary phase and decline in rich media, marked by *CQS1* that encodes *Cryptococcus* quorum sensing protein Qsp1 (Fig S1E, Table S1). *CQS1* is the most abundant transcript in cells in stationary phase conditions and RPMI media, as previously noted [(27)](https://paperpile.com/c/bq2XYM/wSnJ). This cluster of stationary-phase upregulated genes are also enriched in catabolic process functions (GO:0006091 generation of precursor metabolites and energy; GO:0005975 carbohydrate metabolic process), indicating that cells are starving.

A second cluster of 60 co-regulated genes are upregulated at 37°C in both media, marked by *SSA1,* encoding a heat shock protein (Fig S1E, Table S1). This cluster of genes is enriched in protein folding chaperones (GO:0006457, protein folding), including *HSP40, HSP60, HSP70, HSP90,* and *HSP104* family members, as expected from the conserved heat shock response that has been previously observed in *Cryptococcus* [(89)](https://paperpile.com/c/bq2XYM/RtuP). A relatively small number of genes - 60 in this cluster - were unambiguously induced by increased temperature in both media (Table S1; and DGE analysis in Table S2).

A third cluster of 57 co-regulated genes is upregulated in RPMI compared to YPD, marked by the virulence-associated transcription factor *GAT201* (Fig S1E, Table S1). This cluster of genes is also enriched in associations with the stress response (GO:0006950, response to stress). These include cell wall-related enzymes (chitin synthase *CHS4*, chitinase *CHI2*, glucan glucosidase *EXG2*, Endoglucanase *LPI9*), as well as genes associated with redox metabolism (catalase *CAT2*, glutathione transferase CNAG_03848). Differential expression analysis of media conditions (Table S2) shows that *GAT201* is one of the most differentially expressed genes after 1 hour at both 37°C (~24-fold, p < 10^-19^) and 25°C (~13-fold, p < 10^-20^). Gat201 acts through other key transcription factors Gat204 and Liv3 [(27)](https://paperpile.com/c/bq2XYM/wSnJ). Consistent with *GAT201* induction 10 minutes after inoculation, we later observed induction of *GAT204* and *LIV3* expression in RPMI at 30 minutes and onwards (Fig S3).

A fourth cluster of 25 co-regulated genes is even more induced in RPMI, marked by *TSA3*, encoding a thiol-specific antioxidant protein, which is over 80-fold induced (Fig S1E). *TSA3* has been reported to be strikingly induced by temperature and hydrogen peroxide when grown in YNB media [(90)](https://paperpile.com/c/bq2XYM/e7Ik). This cluster is enriched in genes involved in fatty acid biosynthesis (GO:0006629, lipid metabolic process).

Lastly, a large cluster of 321 genes associated with growth is induced in YPD at both temperatures and in RPMI at 25°C only, marked by translation elongation factor *TEF1* (Fig S1E). This cluster of genes is enriched for genes involved in protein synthesis, including ribosomal proteins (GO:0005840, ribosome), translation elongation factors, and amino acid production (GO:0006520, cellular amino acid metabolic process). The cluster is also enriched in genes involved in DNA segregation (GO:0000278, mitotic cell cycle) and mitochondrial biogenesis (GO:0005739, mitochondrion).

**Fig S2: Principal Component Analysis of RNA-seq dataset 1.** Note PC 1 vs 2 panel is a repeat of A1C.


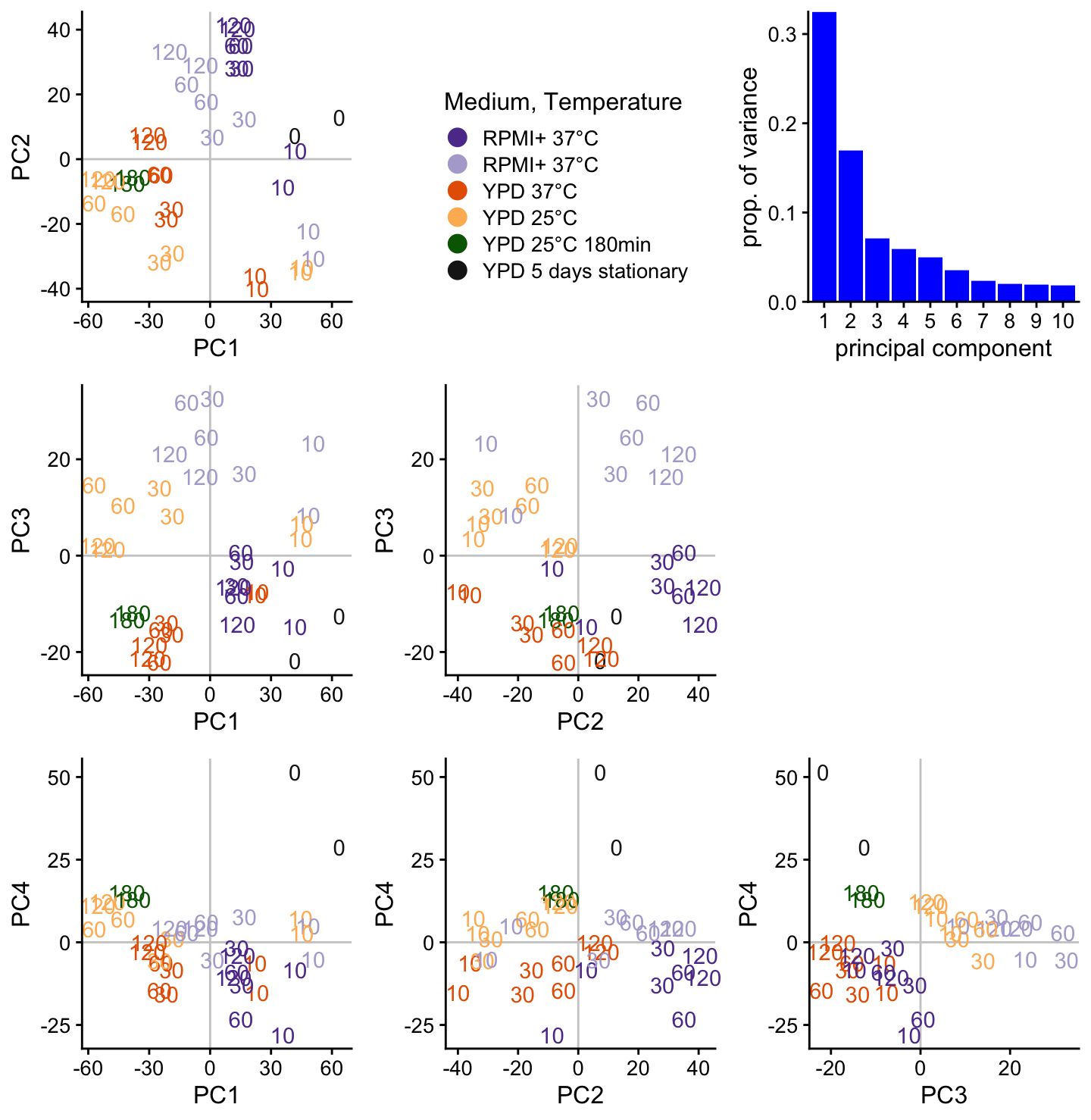


**Fig S3: Thirty representative genes show distinct expression patterns (log2 fold-change per gene).** See Fig S1 legend for details of the experiment.


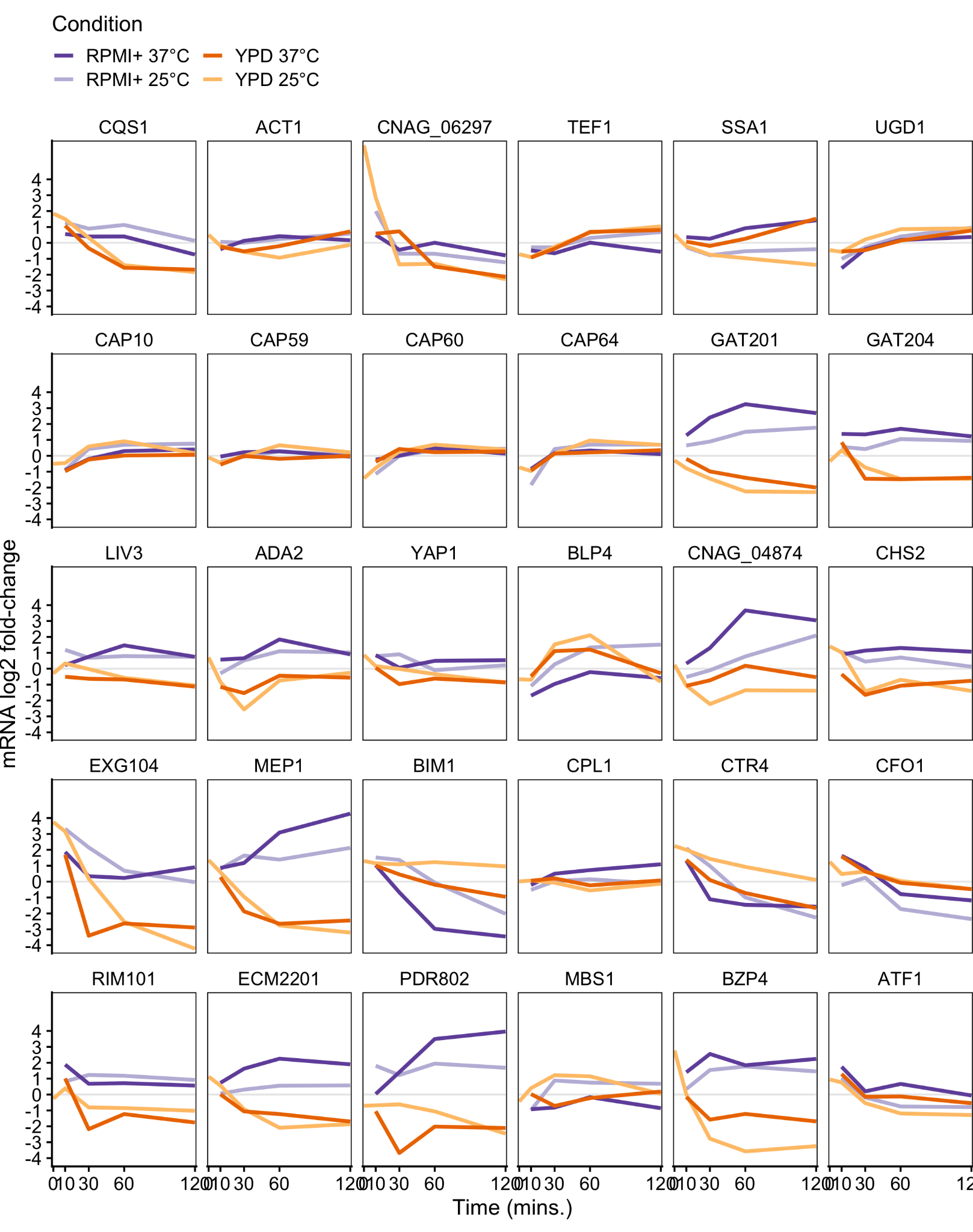


**Additional phenotyping of *GAT201* mutant strains.**

**Fig S4, related to Fig 1: GAT201 promotes capsule biosynthesis and represses budding in RPMI medium (without serum) at 37°C 2 hours after inoculation.** There is no obvious difference in phenotype in rich YPD medium. Micrographs show GAT201 (H99), gat201∆m, and complemented GAT201-C1 strains, stained with India Ink. Scale bar is 10µm.


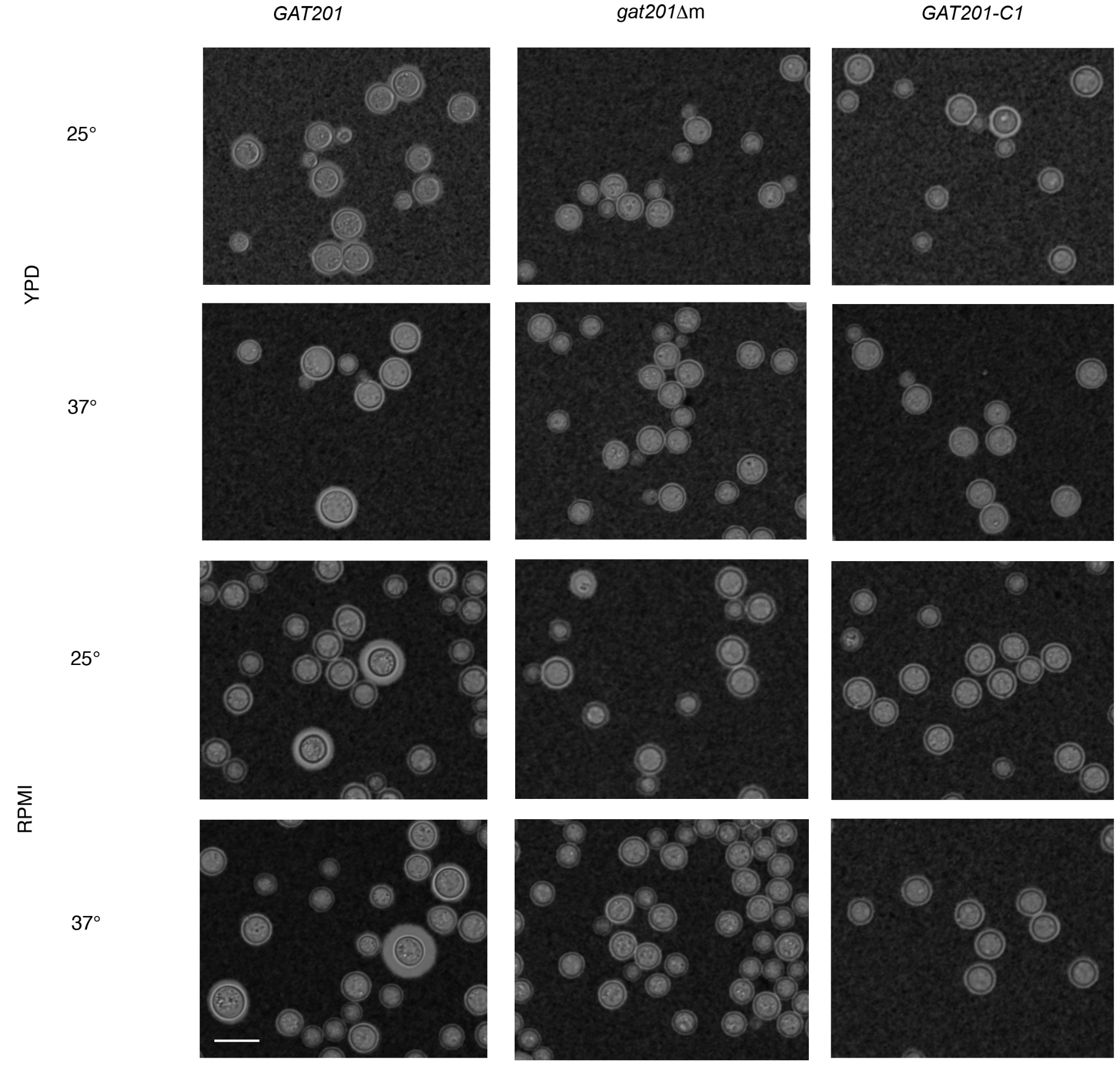


**Fig S5, related to Fig 1: Partial complementation of Gat201 mRNA expression shown by RT-qPCR of GAT201, GAT204, and LIV3 genes compared to 3 reference genes (ACT1, GPD1, SRP14).** In complemented strains, GAT201 mRNA abundance is roughly 10x lower (log2 fold-change ~3.3) than in wild-type *GAT201* (KN99alpha). Cultures were inoculated from overnight growth in YPD and grown in RPMI at 37°C for 7 hours. The figure shows log2 fold-change (∆∆Cq) values from 3 biological replicates (median of 3 technical replicates), and a mean value across the biological replicates. The low value of GAT201 detected in *gat201∆m* represents background, and was only detected at all in 2 out of 3 biological replicates.


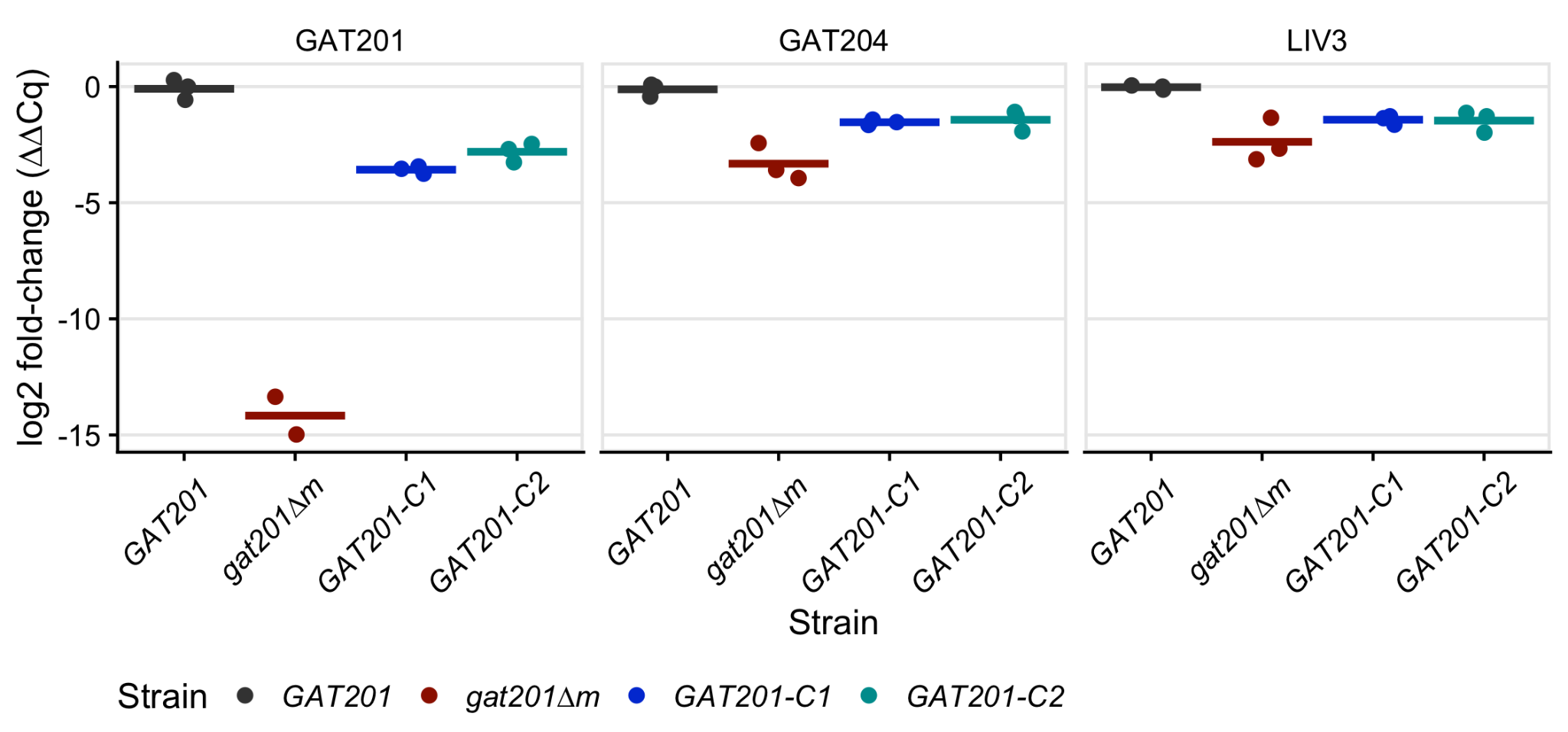


**RNA-seq timecourse on GAT201 dependence of growth in RPMI medium**

We first checked the effects of serum on capsule and budding in *GAT201* (KN99alpha) and *gat201∆m*. Microscopy confirmed that the major phenotypes are serum-independent: *GAT201* cells produce capsule and few buds in both RPMI and RPMI + serum, while *gat201∆m* produce less capsule and more buds in both conditions (Fig S6A, Fig S7).

**Fig S6: Serum is not the dominant driver of *GAT201*-dependent phenotypes in RPMI 1640 media.** A, *GAT201* promotes capsule biosynthesis and represses budding in RPMI medium both with and without serum at 37°C, 2 hours after inoculation. Strains are *GAT201* (KN99alpha) and *gat201∆m*. B, Time from inoculation dominates the overall variance in gene expression regardless of serum addition or *GAT201* allele, shown by principal component analysis on the regularized log-counts per gene in every replicate. C, Only a small set of genes are differentially regulated by serum or by GAT201, shown by clustered heatmap of log2 fold-change per gene calculated by DESeq2. D, Representative gene expression patterns (log2 fold-change).


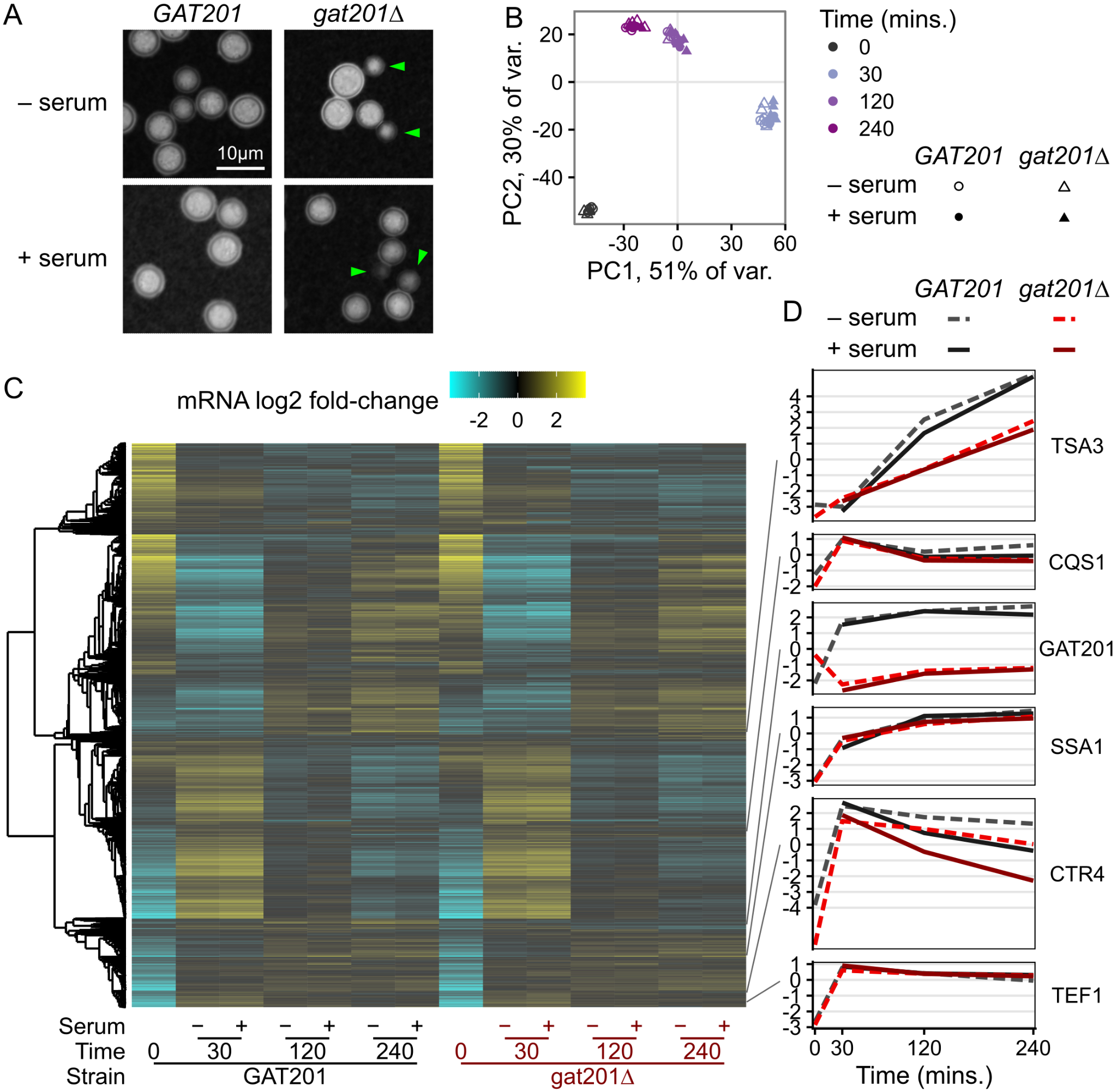


**Fig S7, related to Fig S6A: *GAT201* promotes capsule biosynthesis and represses budding in RPMI medium both with and without serum at 37°C, 2 hours after inoculation.** Strains are *GAT201* (KN99alpha) and *gat201∆m*, here a wider field of view is shown than in Fig S6A.


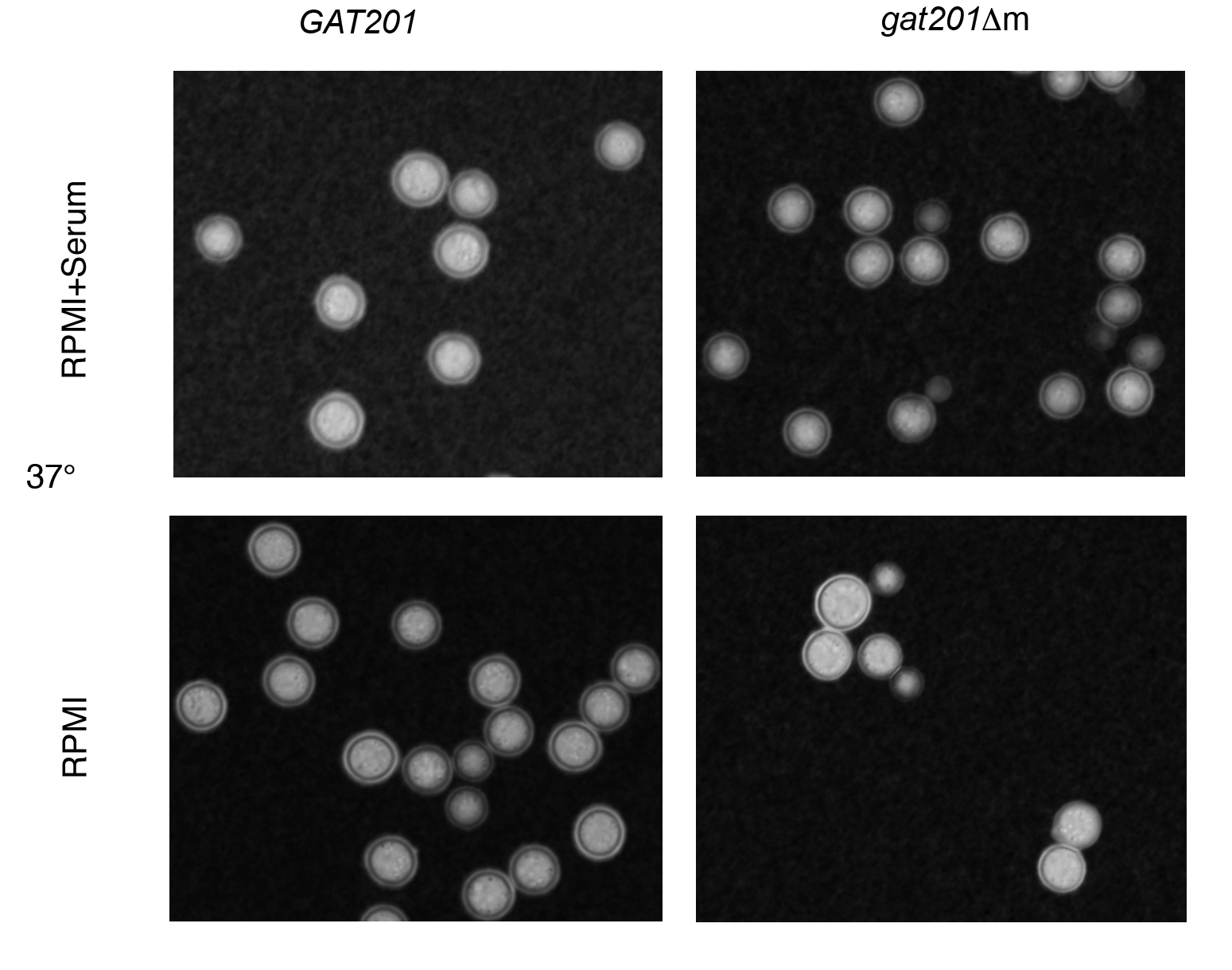


We carried out RNA-seq of *GAT201* and *gat201∆* strains reactivating, from stationary phase, in RPMI and RPMI + serum at 30 min, 2 hrs, and 4 hrs. Principal Component Analysis on the regularized log-counts per gene revealed that time after inoculation is the dominant driver of variance in this dataset (Fig S6B). 81% of variance is attributed to the first two principal components, in which datasets cluster together at each timepoint from all strains grown with and without serum. Principal component 3 (4% of variance) distinguishes between *GAT201* and *gat201∆* strains (Fig S8), indicating that the RNA-seq detected a specific GAT201-dependent transcriptional program, that we return to later. We were surprised not to detect a stronger serum-dependent effect, given previous work on the effect of serum on growth and capsule production in *Cryptococcus*, although this may reflect the short 4 hour time course [(91)](https://paperpile.com/c/bq2XYM/CN0A).

Consistent with serum not being the major driver of phenotype, we found few differences in the overall transcriptome-wide response to serum (Fig S6C, Table S3). Transcriptome-wide expression evolves over time with a large cluster of genes that are strongly upregulated at 30 minutes and still up at 2 and 4 hours. Conversely, a large cluster that is strongly downregulated at 30 minutes becomes less repressed at later timepoints. Expression of a set of representative genes selected from Fig 1 shows similar patterns to the previous dataset (Fig S9). *TSA3* is strongly induced over time in RPMI and RPMI + serum, dependent on *GAT201* status. *CQS1* is highly expressed at 0 minutes and further induced in RPMI and RPMI + serum. *GAT201* itself is induced in RPMI and RPMI + serum in *GAT201* cells. The RNA-seq protocol used here is a 3’-end targeted assay that detects a transcript fragment of *GAT201* RNA in the two *gat201∆* strains that have a truncated or deleted coding sequence; these transcript fragments do not encode a functional Gat201p. Both *SSA1* and *TEF1* are induced in RPMI and RPMI + serum as cells reactivate protein synthesis.

A small set of genes in wild-type cells differentially respond to the presence of serum at all time points. For example, copper transporter *CTR4* (Fig S6D) and copper starvation-induced membrane protein *BIM1* (Fig S9) are induced by 30 minutes growth in all media, and lower induction in media with added serum suggests differences in copper ion availability. Differential gene expression analysis with DESeq2 detected under 100 each of serum-upregulated and serum-downregulated genes at 5% FDR with at least 2-fold change, in *GAT201* strains (Fig S11, Table S3). Loss of *GAT201* dampened this response to serum, with about 4-fold more DEGs detected in *GAT201* than in *gat201∆* (Fig S11). This dampened response in *gat201∆* strains corroborates previous studies that found an association between expression of *GAT201* and downstream targets in alternative DMEM media with serum [(23)](https://paperpile.com/c/bq2XYM/8Rzj).

**Fig S8, related to Fig S6B: Principal Component Analysis of RNA-seq dataset 2, comparing *GAT201* (wild-type) to *gat201∆* strains.** Note PC 1 vs 2 panel is a repeat of 3B.


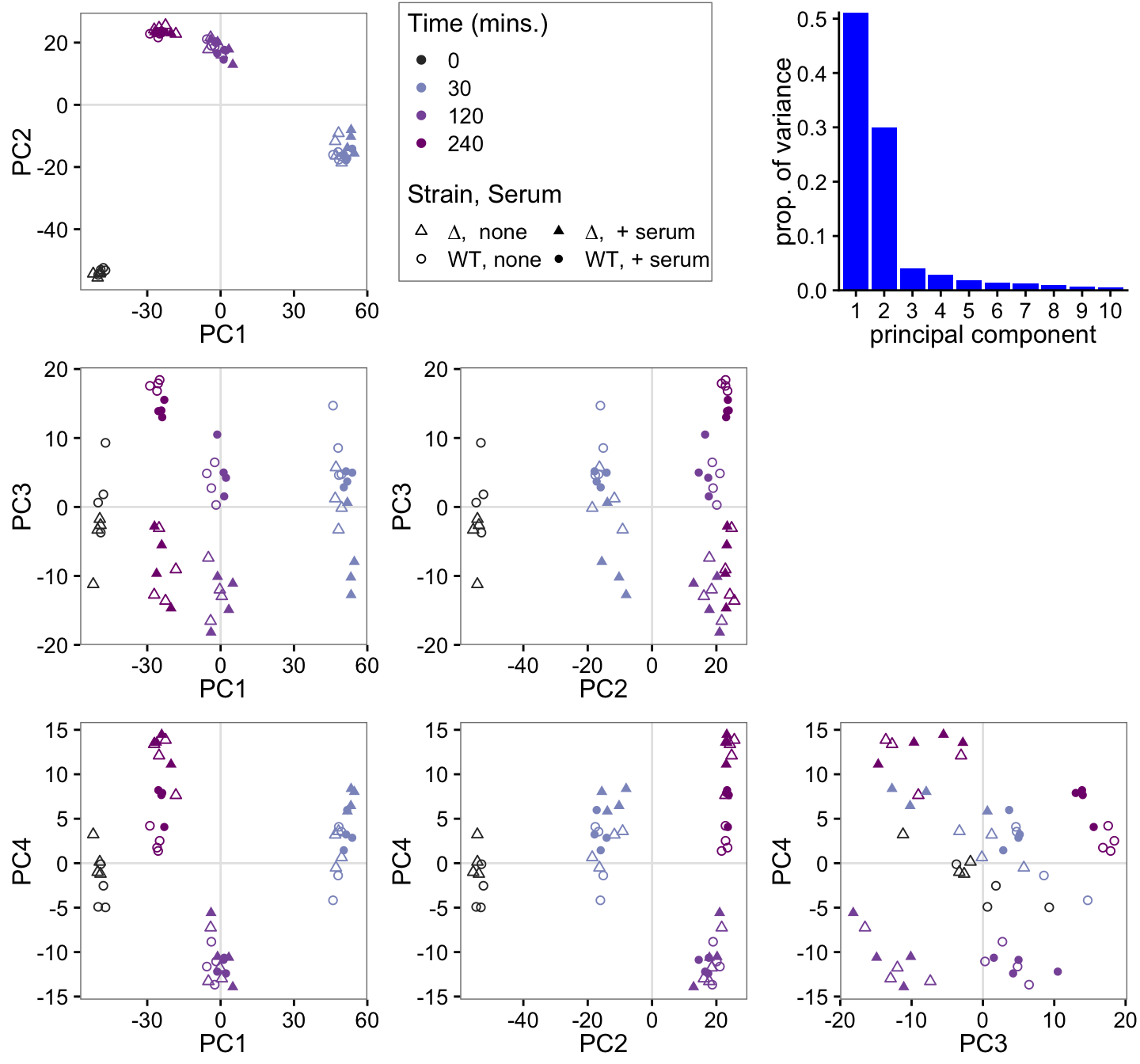


**Fig S9, related to Fig S6D: Thirty representative genes show distinct expression patterns and GAT201 dependence, again in log2 fold-change per gene.** See Fig S6 legend for details.


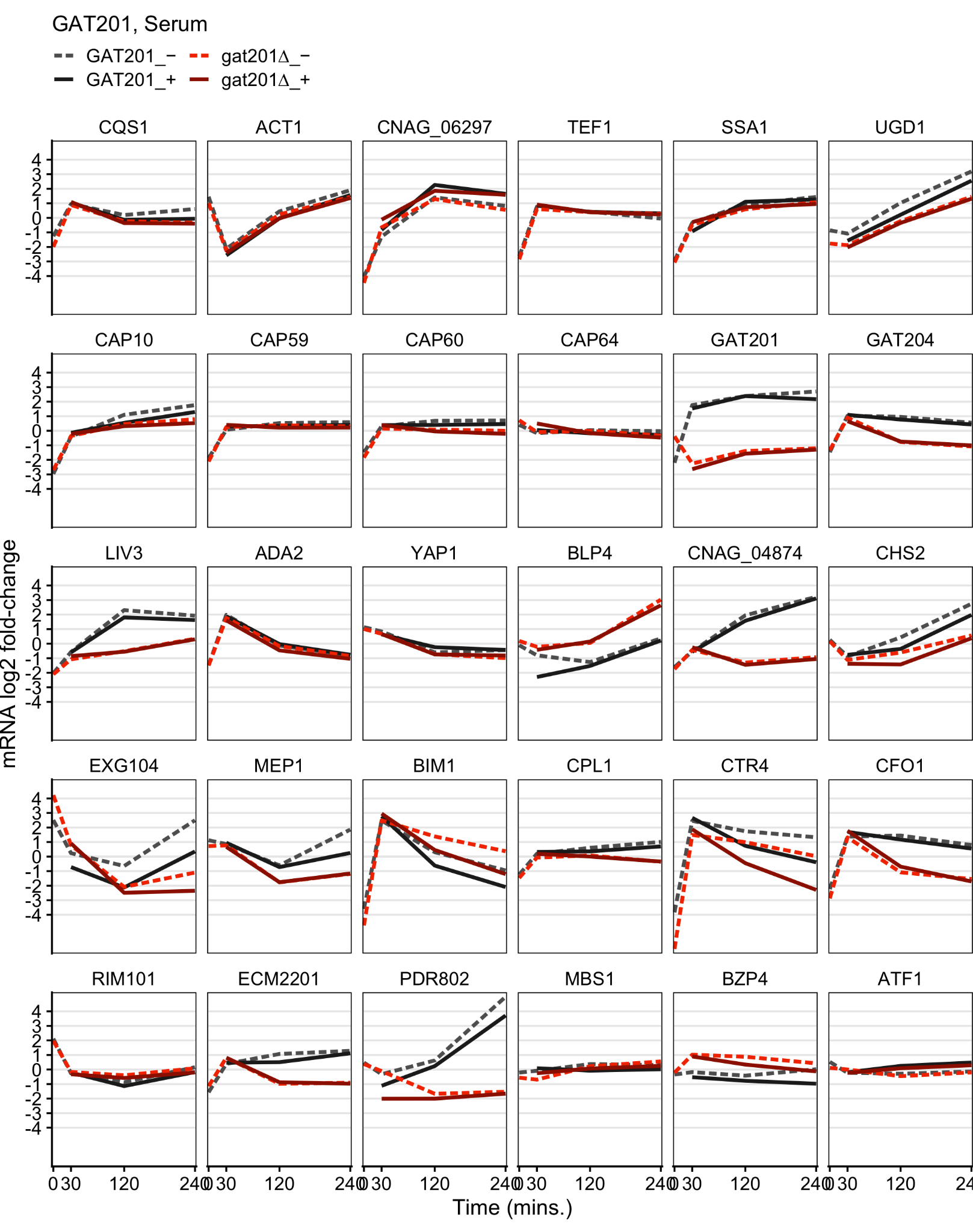


**Comparison of RNA-seq datasets**

Comparing our two RNA-seq datasets presented here indicates that the signature of *GAT201* pathway activation is consistent. Direct comparison of differential gene expression between the matched conditions of 0 minutes (pre-inoculation) and 2 hours RPMI + serum in wild-type cells shows moderate correlation, R = 0.27 (Fig S10, Table S2, Table S3). The major trends that are the focus of this study are consistent: *GAT201* and its targets are induced in RPMI + serum, as are many genes associated with protein synthesis. Despite this, there are some differences that may reflect local experimental conditions. For example, we observe that copper-dependent genes *CTR4* and *BIM1* are induced in RPMI in dataset 2 and not in dataset 1. This may reflect that the experiments were performed years apart by different experimentalists on different campuses. The datasets were also collected using different RNA-seq approaches. For dataset 1, we used RNATagSeq and rRNA depletion for full-length mRNA sequencing. For dataset 2, we used QuantSeq FWD for 3’-targeted mRNA sequencing.

**Fig S10: Comparison of differential expression in dataset 1 (Wakeup) and dataset 2 (Gat201), differential expression between 0 hours (pre-inoculation timepoint) and 2 hours in RPMI + serum for wild-type strain.** The lowest 10% expressed transcripts in both of these datasets (by baseMean from DESeq2) were filtered out. 30 genes shown in Figs S3, S10 and discussed in the text are highlighted.


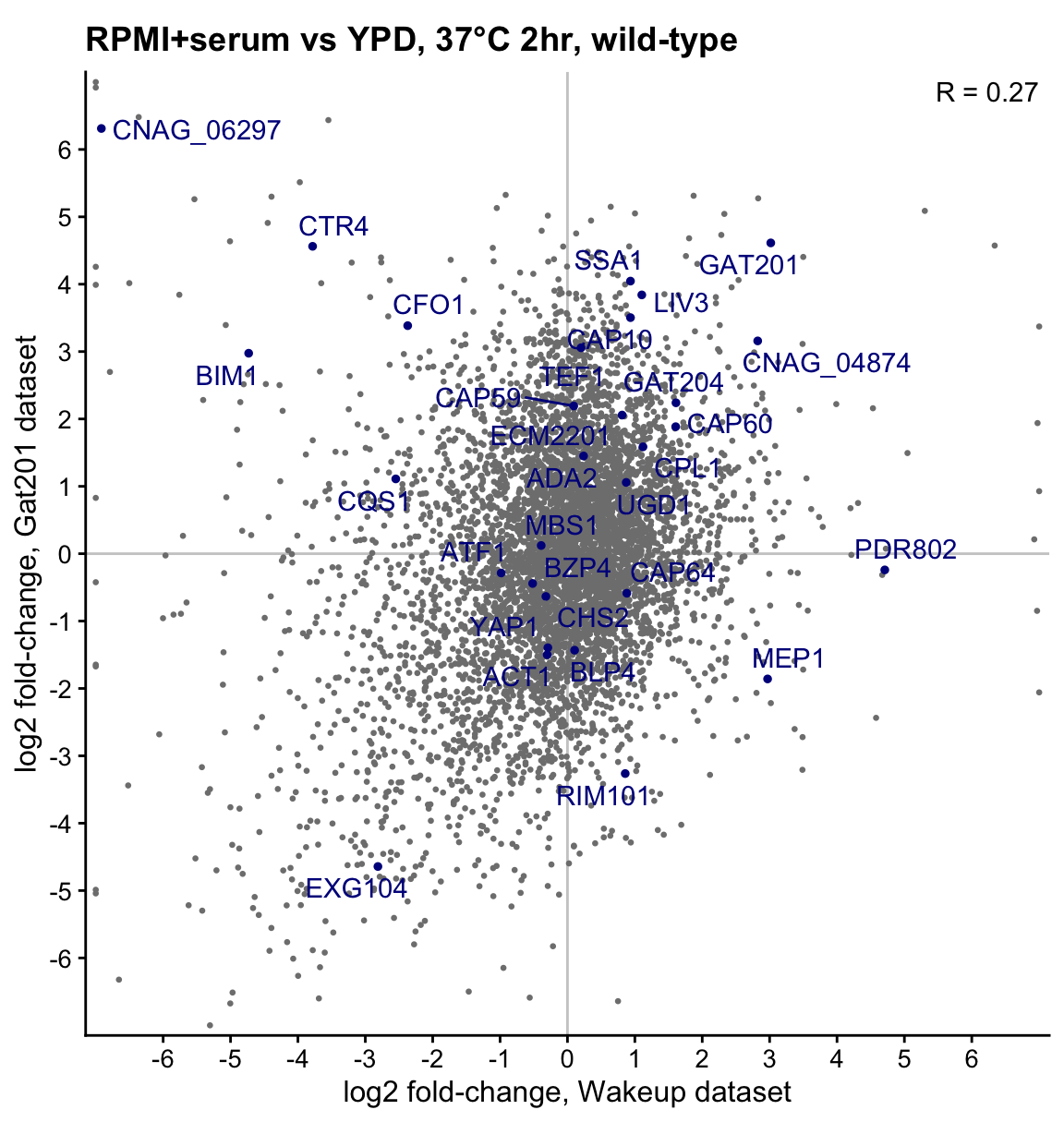


**Fig S11, related to Fig S6. Serum affects expression of a small set of transcripts.** A, Number of differentially expressed genes (2x changed, 5% FDR) dependent on serum at each timepoint in *GAT201* and *gat201∆* strains. B, Serum-dependent log2 fold-change and p-values in *GAT201* at 4 hours. C, Serum-dependent log2 fold-change and p-values in *gat201∆* at 4 hours.


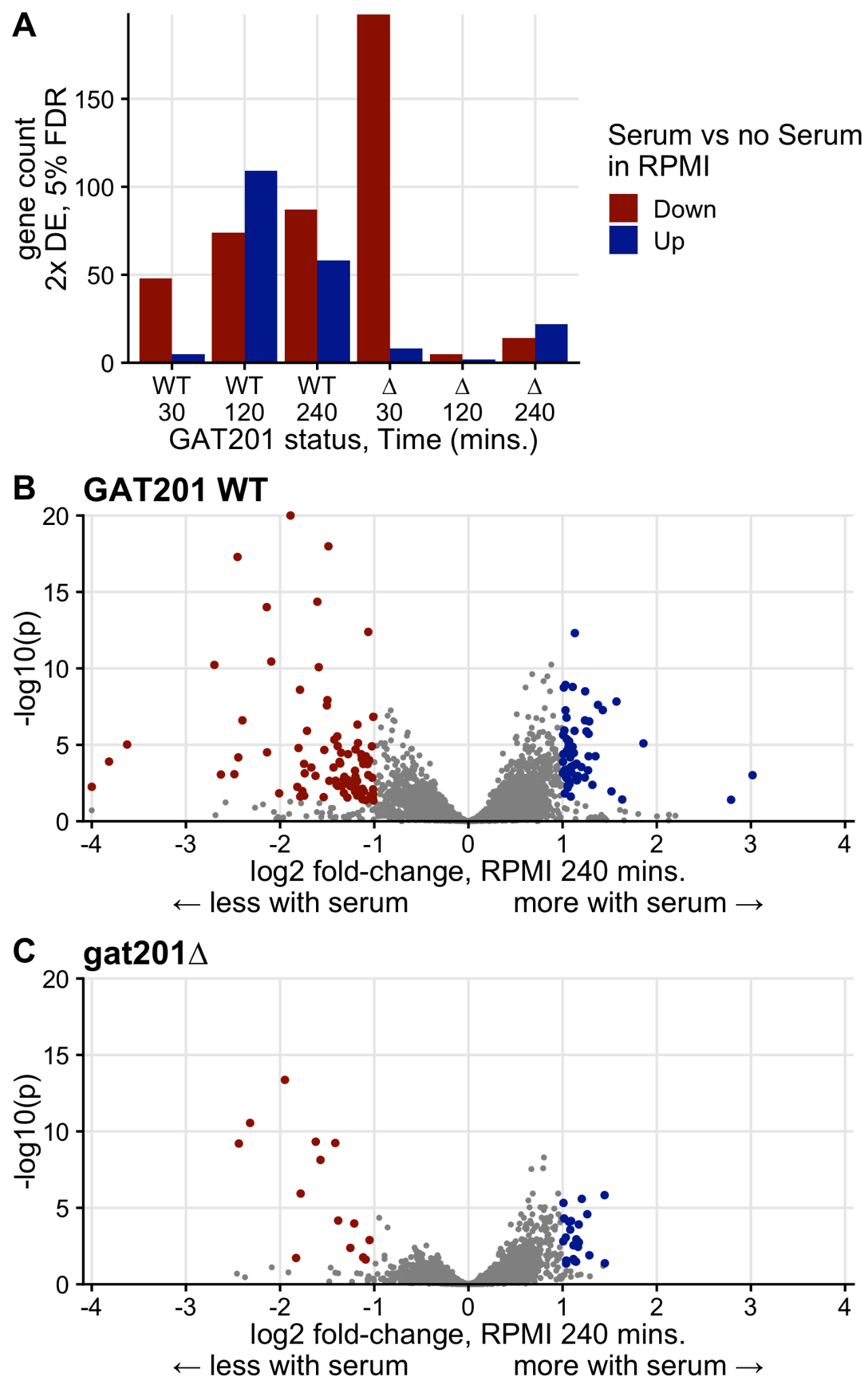


**Most GAT201-dependent DEGs are direct targets of GAT201.**

A few hundred genes were found to be dependent on the expression of *GAT201* across the timecourse. After 5 days of growth in YPD (0 minute timepoint), there were only 82 2-fold differentially expressed genes at 5% FDR, up in wild-type *GAT201* strain compared to mutant *gat201∆*. GO analysis suggested enrichment for upregulated genes involved in microtubule-based movement (GO:0007018), and lipid metabolism (GO: 0006629). 35 genes were down in *GAT201* compared to *gat201∆* cells, with GO analysis highlighting probable changes in the cell wall (GO: 0016798, hydrolase activity acting on glycosyl bonds; GO:0005975, carbohydrate metabolic process) including the exoglucanase *EXG104*. However, after 30 minutes of incubation in RPMI or RPMI+serum, the *GAT201*-dependent differentially expressed genes are largely distinct from those observed in stationary phase. For example, *EXG104* is upregulated in *GAT201* cells after reactivation. The number of 2-fold differentially expressed genes at 5% FDR between *GAT201* and *gat201∆* increases from 30 minutes up to 240 minutes (Fig S12A). We observed over 200 differential-expressed genes in each direction in RPMI (without serum) at 240 minutes (Fig S12B). Also, the magnitude of each gene’s differential expression between strains tends to increase over time (Fig S12C). Below, we focus on analysis of the RPMI 240 minutes timepoint.

Loss of activation of the Gat201 pathway in *gat201∆* strains is confirmed by lower expression of known Gat201 targets during reactivation including *BLP1* (Fig S12C), *GAT204*, and *LIV3* (Fig S9). Functional enrichment analysis of genes up in *GAT201* cells highlighted changes at the cell surface, including transmembrane transport (GO:0055085) and cell-wall related terms carbohydrate metabolic processes (GO:0005975) and glycosyl hydrolase activity (GO:0016798). Metabolic pathway analysis highlighted *GAT201*-upregulated genes required for capsule biosynthesis (PWY-5114__PK__MetaCyc, UDP-sugars interconversion), including UXS1/CNAG_03322 and UGD1/CNAG_04969 [(14)](https://paperpile.com/c/bq2XYM/FsDc), consistent with the observed *GAT201*-dependent capsule synthesis during reactivation. Genes that are down in *GAT201* strains are enriched for ribosome biogenesis (GO:0042254) and related terms, indicating downregulation of the core biosynthetic process of protein synthesis. Genes down in *GAT201* strains were also enriched in carbohydrate metabolic processes (GO:0005975), indicating that different sets of carbohydrate-processing functions are upregulated and downregulated depending on *GAT201*. Overall, this implicates Gat201 in the regulation of carbohydrate metabolism and cell surface remodeling, and in repressing core biosynthetic processes related to growth.

We found that many of the *GAT201*-dependent genes are direct targets of Gat201. Previous measurements of binding of Gat201 to DNA by ChIP-seq found roughly 1200 enriched peaks out of approximately 6800 annotated cryptococcal genes [(27)](https://paperpile.com/c/bq2XYM/wSnJ). We compared these targets to our list of differentially expressed genes at 240 minutes in RPMI (Fig S12D). Over 50% of the *GAT201*-upregulated genes here are direct targets (151/290), representing approximately 3x enrichment. The *GAT201*-downregulated genes at 240 minutes in RPMI are also about 2x enriched in direct targets (73 direct targets / 210 downregulated genes). We see similar enrichment at earlier timepoints.

**Fig S12. GAT201 specifically affects gene expression as cells reactivate, acting via its direct targets.** A. There is more GAT201-dependent differential gene expression at later time points in activation. Fig S12A shows the number of 2-fold differentially expressed (DE) genes at 5% FDR at each combination of growth condition and time point. Differential expression is calculated by DESeq2 using the Wald test as the average over 4 samples: 2 wild-type and 2 deletion strains, each strain measured in biological duplicate. B. GAT201 promotes upregulation of specific genes more than downregulation. Volcano plot of log2 fold-change and p-value, with differential expressed genes calculated and coloured as in panel A. Genes with extreme p-values or fold-changes are plotted at the edge of the panel area. C. GAT201-dependent differential gene expression is more extreme at later timepoints. The panel shows all the genes that are at least 8x differentially expressed in any combination of condition and time, ordered by their average fold-change at 4 hours. D. Over half of the upregulated differentially expressed genes are direct targets of GAT201. Venn diagram shows the number DEGs in RPMI at 4 hours (as in panel B) compared to Gat201 targets measured by ChIP-seq from Homer et al. 2016. This is approximately a 3-fold enrichment.


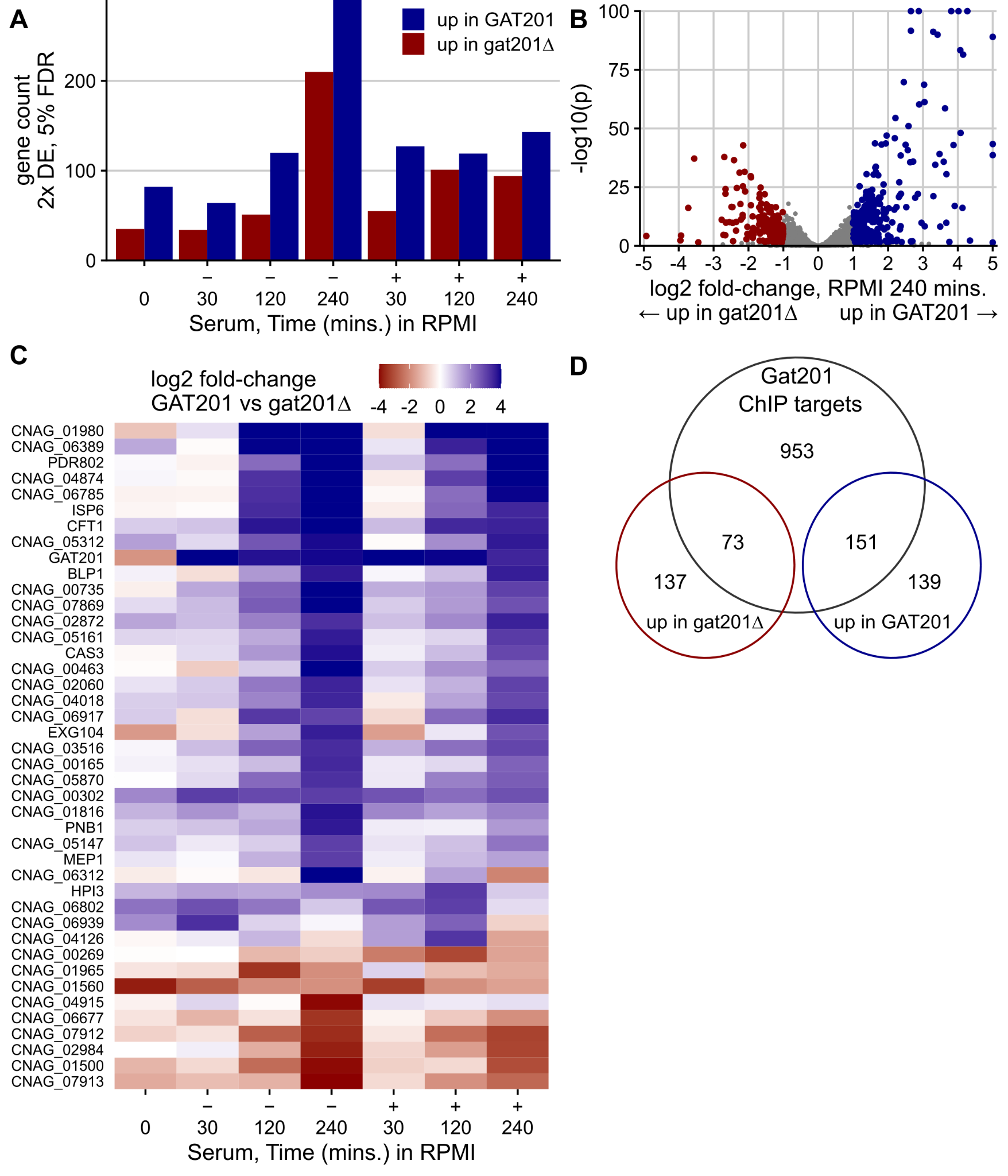


**Fig S13, related to Fig 4: Growth curves of mutants in the Gat201 pathway.** Panels A,B,C show growth curves, all 4 biological replicates in 3 technical replicates, of OD595 corrected for values of blank wells. Individual wells are plotted as faint lines, and smoothing spline for each strain plotted as thick lines. The summaries from panel A are also shown in Fig 2.


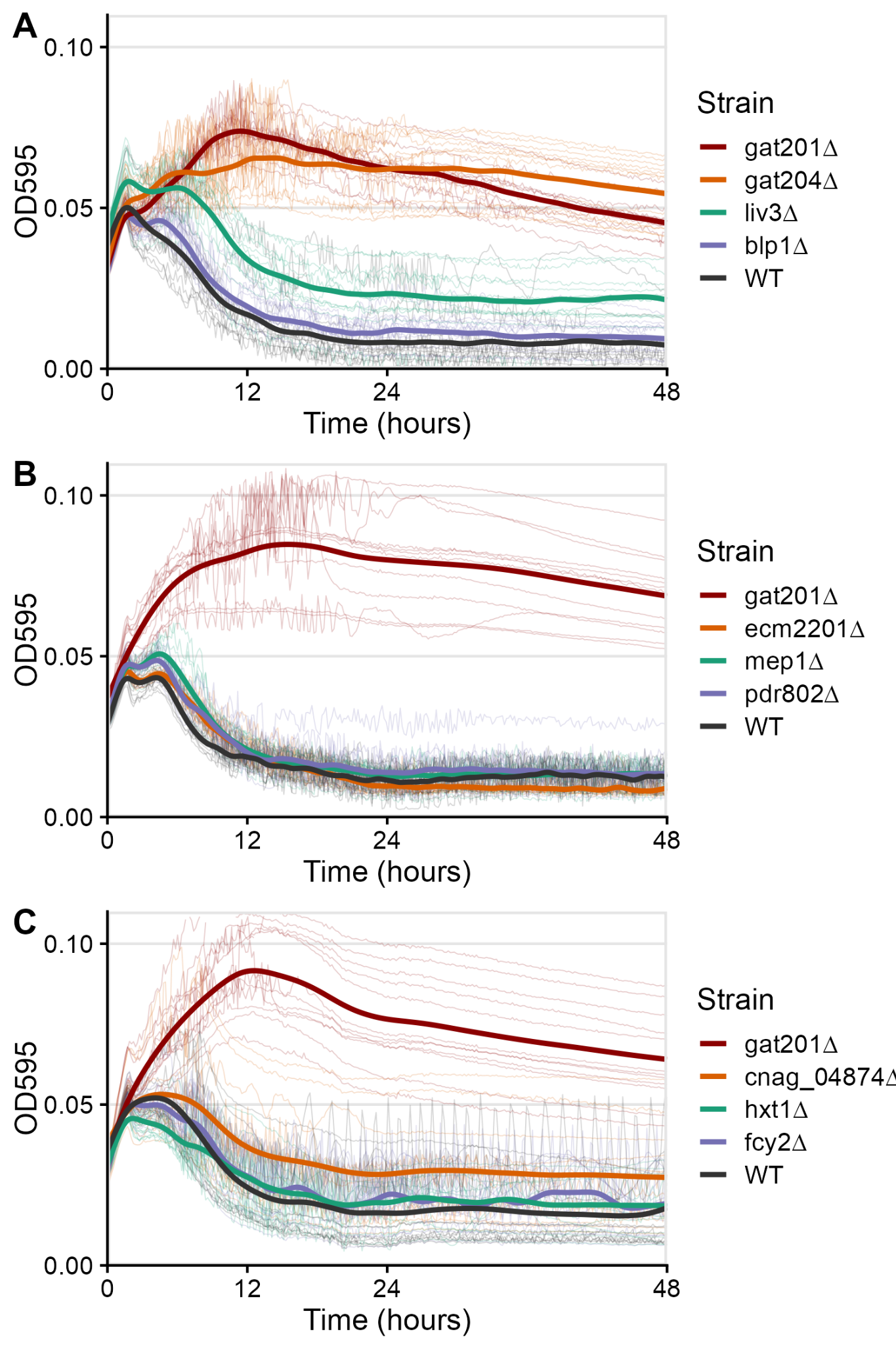


**Fig S14, related to Fig 4: The double mutant gat201∆ liv3∆ has similar phenotypes to single mutants of partially permitting growth in RPMI.** Panel shows growth curves, 3 biological replicates in 3 technical replicates, of OD595 corrected for values of blank wells. Individual wells are plotted as faint lines, and smoothing spline for each strain plotted as thick lines.


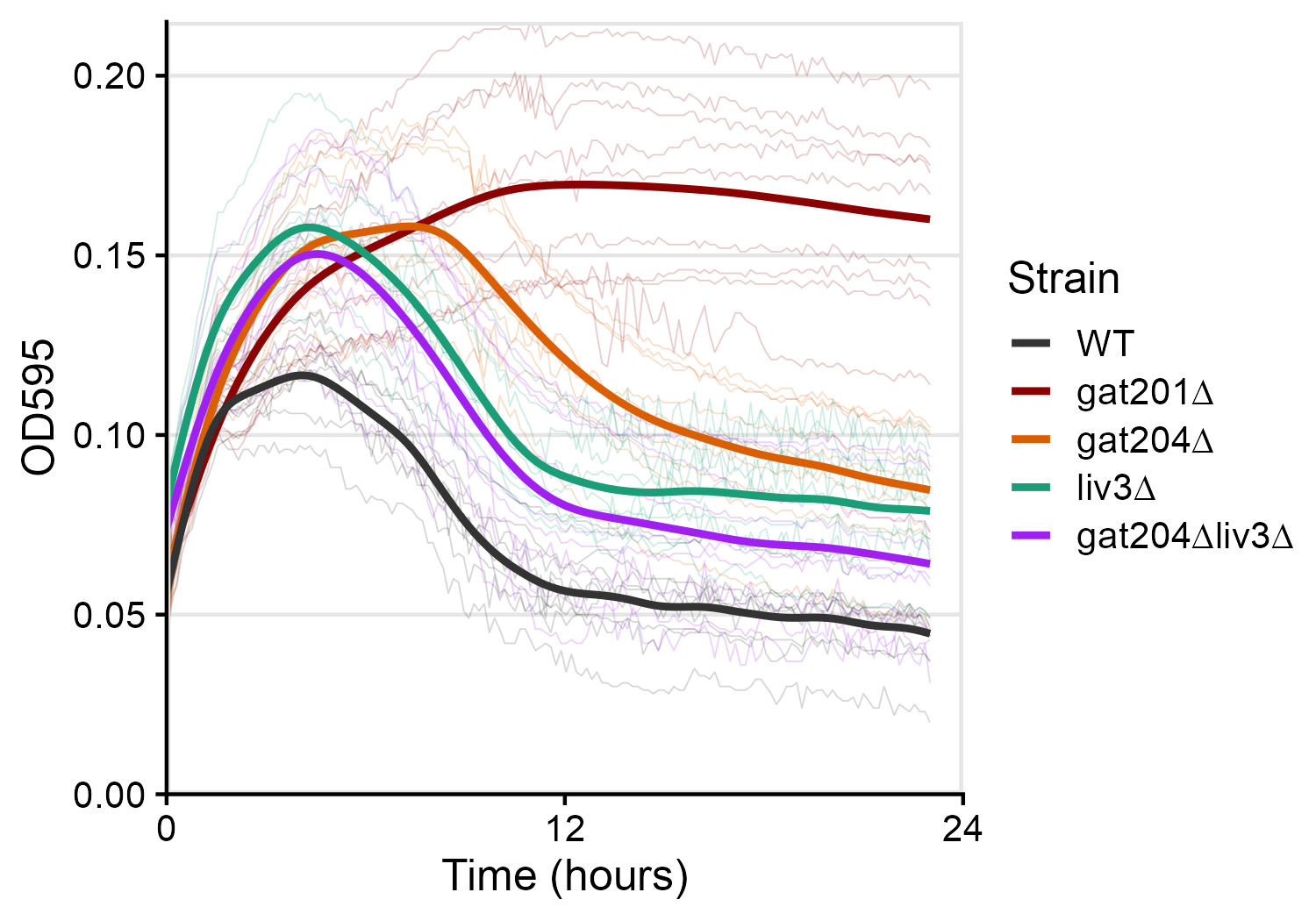


**Fig S15: GAT201 has a minor effect on growth in media buffered to near-neutral pH with mono and dibasic sodium phosphate and β-glycerophosphate.** Cells were grown as in Fig 2C and their OD595 measured, but in Gibco™ CO_2_-independent media. Individual wells are plotted as faint lines, and smoothing spline for each strain plotted as thick lines.


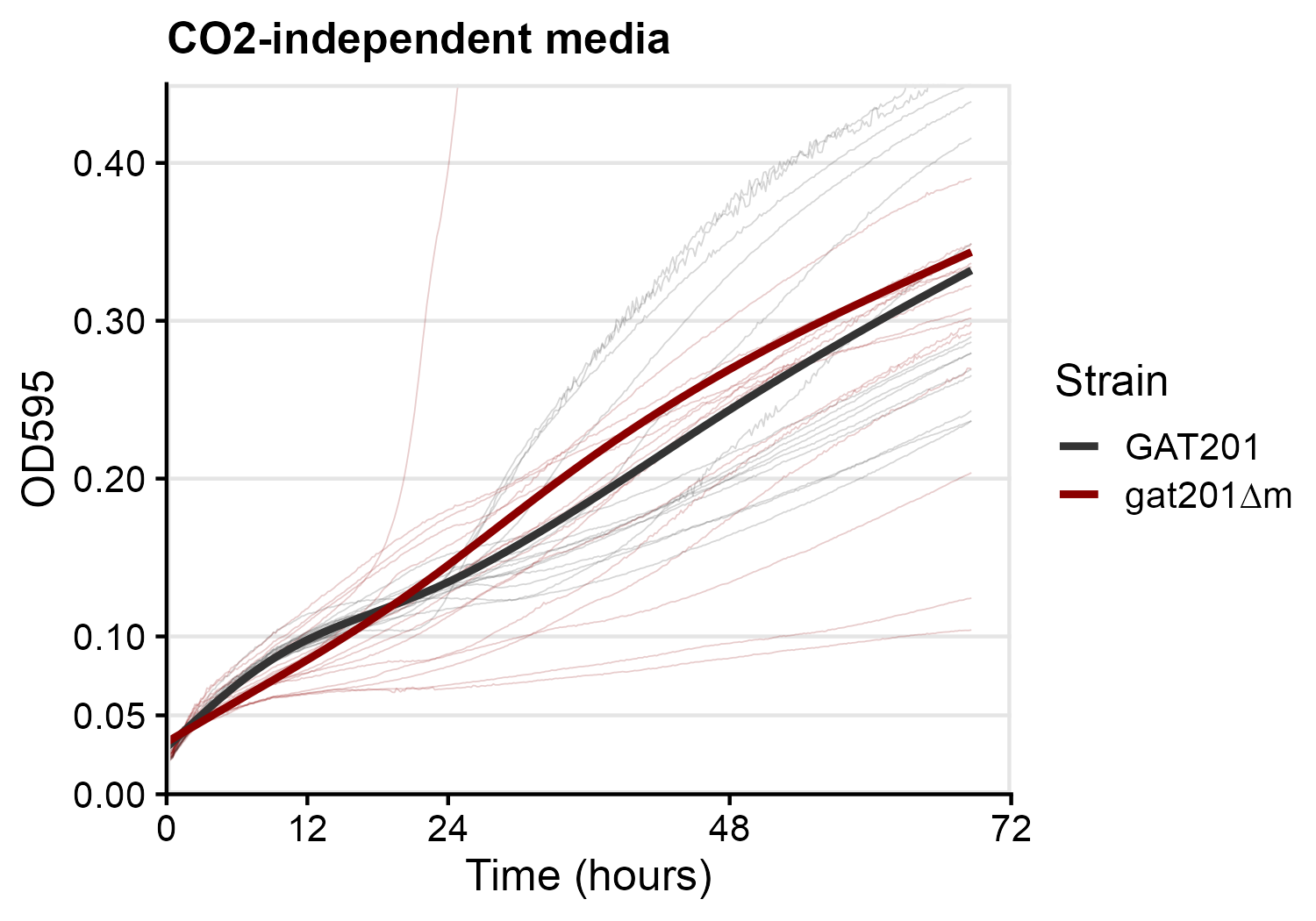


**Fig S16, related to Fig 3: The effect of *GAT201* on growth depends on sodium bicarbonate (NaHCO_3_)**. Panel shows growth curves, each column showing a single biological replicate (Biol. Rep.), and within those 3 technical replicates for each strain, of OD595 corrected for values of blank wells. Individual wells are plotted as faint lines, and smoothing spline for each strain plotted as thick lines. The summary splines for biological replicate 3 are also shown in main Fig 3.


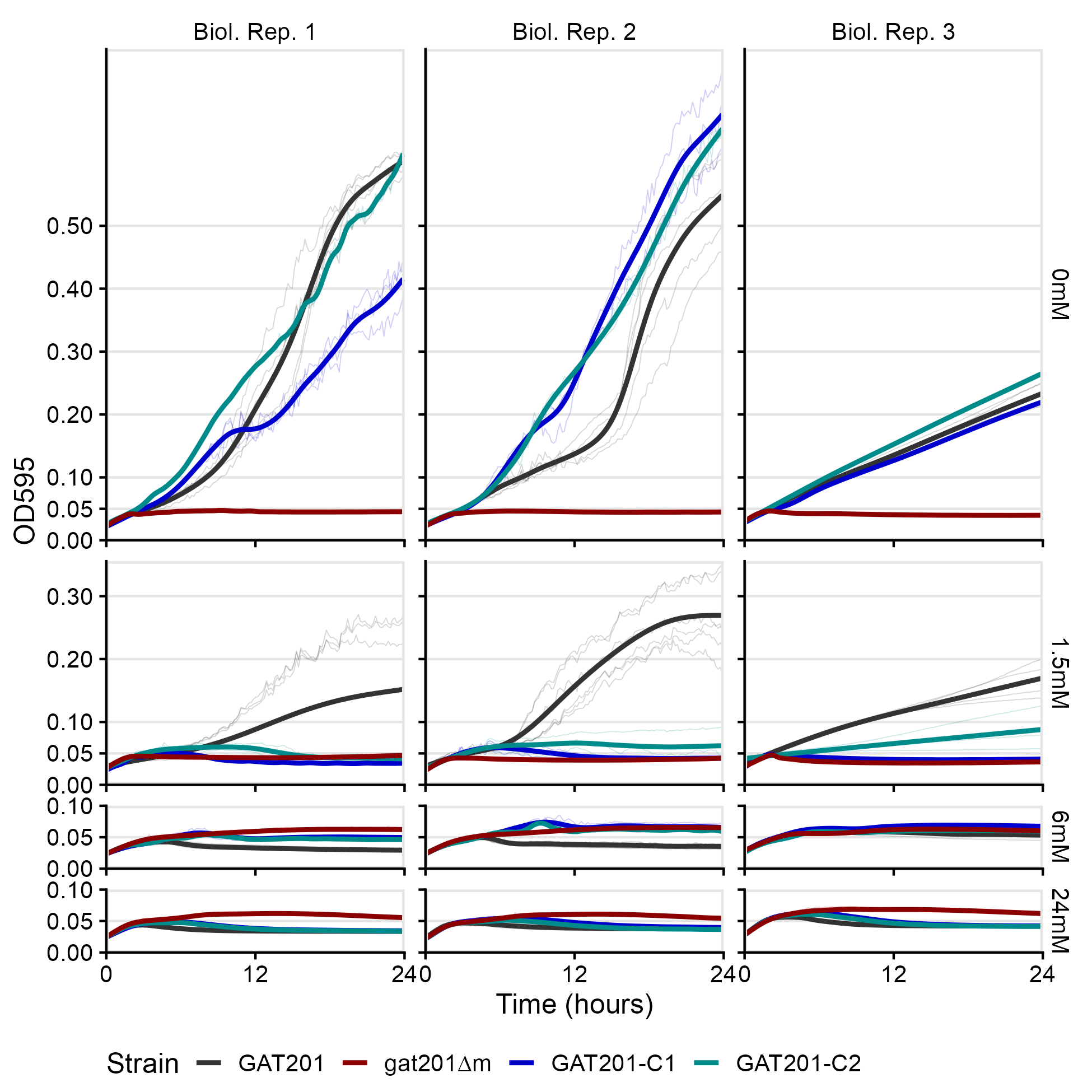


**Fig S17: Growth phenotypes due to sodium bicarbonate (NaHCO_3_) and GAT201 but not dependent on cyclic AMP (cAMP).** Fig shows growth curves of wild-type *GAT201* and deletion mutant *gat201∆m*, grown in RPMI with no added NaHCO_3_ (top row, “−”) or 24mM NaHCO_3_ (“+”), and with either mock H_2_O addition, exogenous cAMP (N6-2'O-dibutyryl-cAMP; dbcAMP) to stimulate the pathway, or sodium butyrate (Nabut) as an additional control.


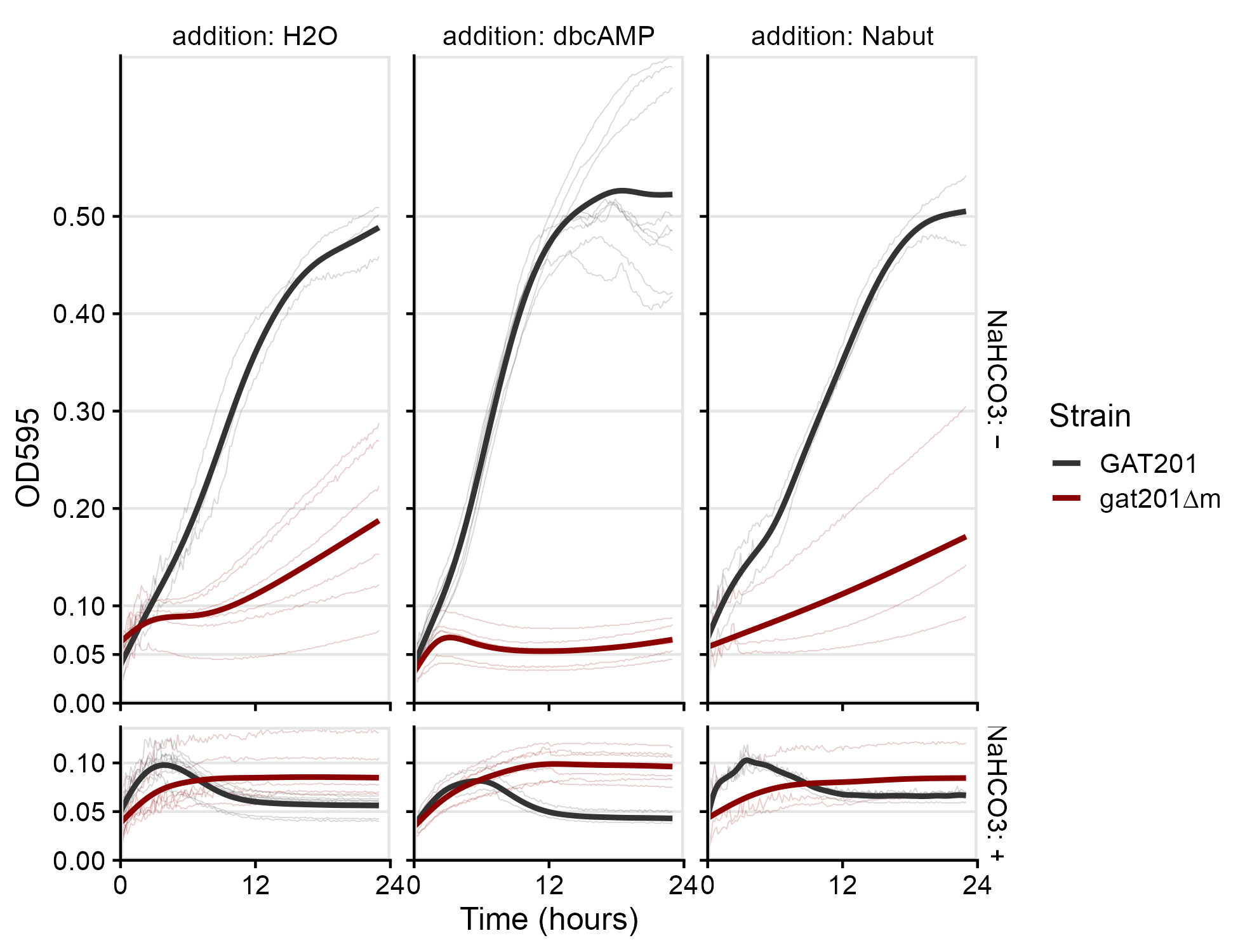


**Extended analysis of *GAT201* homology**

**Fig S18: Extended phylogenetic analysis of Gat201 and related GATA-domain zinc finger proteins shows that fungal *C. neoformans* Gat201, *C. albicans* Brg1, *A. nidulans* NsdD, N. crassa *sub-1*, and amoebal *D. discoideum* gtaI, form a subfamily.** A, Phylogenetic tree calculated by IQ-TREE from a full-length protein alignment by MAFFT, highlighting the groups of *C. neoformans* Gat201-like, *S. cerevisiae* Gat2-like, and *C. neoformans* Gat204-like proteins. B, Multiple sequence alignment by MAFFT of the GATA zinc finger domain from the same proteins in the same order as panel A.


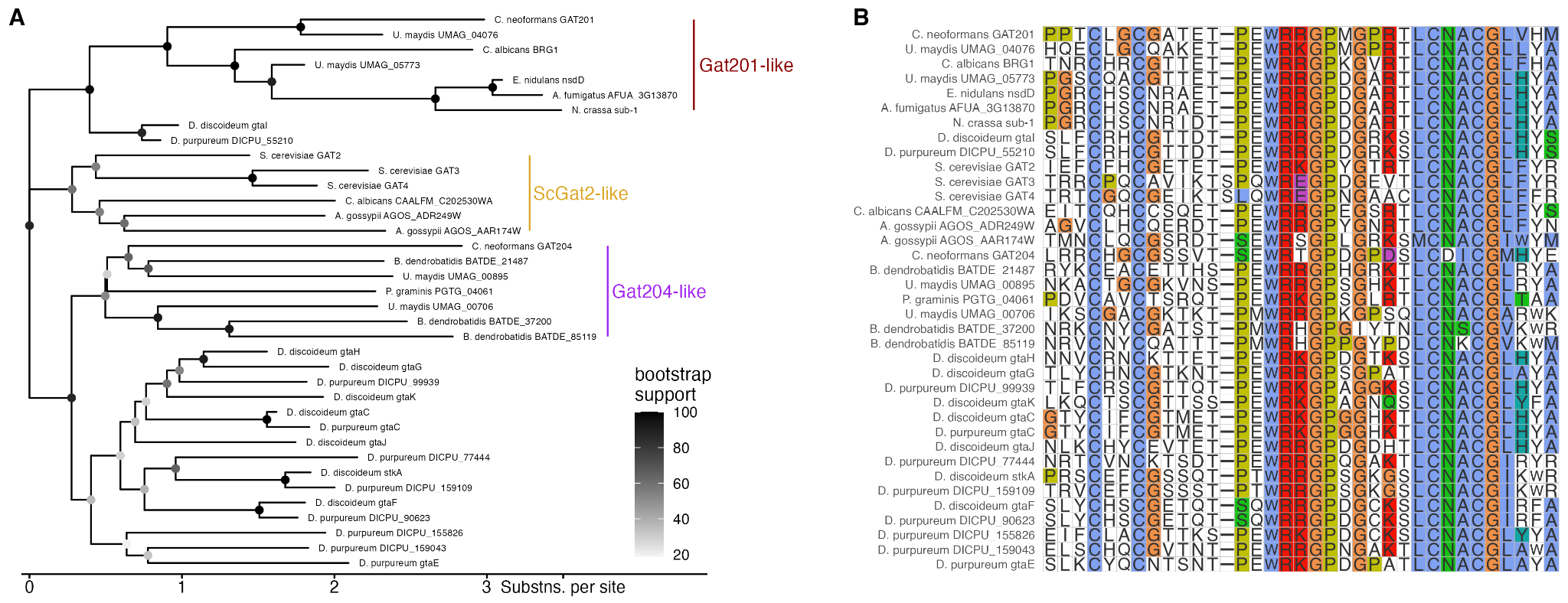


As an alternative method of searching for true homologs of *GAT201*, we looked for syntenic (gene-order conserved) homologs using the GenomicusFungi synteny browser [(37)](https://paperpile.com/c/bq2XYM/16yi). We searched against *C. deneoformans GAT201* (CNC06330), which is syntenic to *C. neoformans GAT201* across the locus. We found a set of basidiomycete syntenic homologs with a C-terminal GATA-like domain, including *U. maydis* UMAG_04076, that was identified as a close homolog in our phylogenetic analysis. Within the Agaricomycotina, the gene neighborhoods of these GATA-like domain proteins were dynamic, with extensive recombination. While there was limited preservation of wider gene synteny, GATA-like proteins within the Basidiomycota tend to be flanked upstream by genes encoding a Vms1-like protein (CNC06290, Ch3, UMAG_04964, Ch15), similar to *S. cerevisiae* YDR049W*,* (ChIV), and a lipid binding protein (CNC06300, Chr3; and UMAG_11290, Chr11) homologous to *S. cerevisiae* YJL036W (ChrX). There is limited longer range synteny downstream of *GAT201*, but the *GAT201* gene is closely flanked by a DEAD box RNA helicase (CNC06360, Chr3; and UMAG_04080, Chr11). *S. cerevisiae* encodes two orthologous genes of the DEAD-box RNA helicase: *DED1* and *DBP1* (YOR204W and YPL119C, located on Chr XV and Chr XVI respectively)*.* Examination of synteny around these genes in *S. cerevisiae* identified genes that are well conserved in *Cryptococcus* and *Ustilago,* including *BEM3* (CNJ02560, Chr10; UMAG_11852, Chr2) and *HIS3* (CNH01620 Chr8; UMAG_11859 Chr2), neither of which were syntenic with GAT201 in either *Cryptococcus neoformans* or *Ustilago maydis,* nor even located on the same chromosome (GAT201: CNC06330, Chr 3; UMAG_04076, Chr 11). Overall, this analysis does not support a syntenic relationship between *GAT201* and other genes containing GATA-like domains beyond the Basidiomycota.

**Appended Materials and Methods**

**Strains, media, and growth conditions**

*RNA-seq Dataset 1*

Wild-type *C. neoformans* H99 were maintained on YPD agar plates at room temperature. For stationary phase, cells were inoculated into multiple tubes of 5 ml liquid YPD (1% yeast extract, 2% Bactopeptone, 2% Dextrose) and incubated for 5 days at 200 rpm, 30°C. On day 5, the temperature was reduced to 25°C and the cells were allowed to adjust for >4 hours. 1mL of this culture was collected for RNA extraction as the “0 minute” timepoint. Cells were counted by hemocytometer at approximately 3.5 x 10^8^ cells. Four aliquots of 3 ml of stationary phase culture were pelleted and resuspended in conditions: 25°C YPD, 37°C YPD (prewarmed), 25°C RPMI 1640 + 10% heat-inactivated fetal calf serum (HI-FCS), and 37°C RPMI + 10% HI-FCS (prewarmed), 100 ml each, so roughly a 1:30 dilution. The precise media used was RPMI 1640 with L-glutamine, sodium bicarbonate and pH indicator phenol red (Sigma R8758). Fetal calf serum (Biosera FB-1285) was heat-inactivated by incubating at 56°C for 30 minutes, then aliquoted and stored at -20°C until required. The pH was checked at time 0 and 120 minutes and confirmed to be within physiological range for the duration of the experiment (7.0 - 8.5). Cells were incubated at 60 rpm. Another stationary phase sample was inoculated into at YPD at 30°C and grown at 150 rpm for 180 min. The two biological replicates were collected on two successive days.

*RNA-seq Dataset 2*

Wild-type KN99a and KN99alpha, and Gat201 mutants from Madhani lab and Bahn lab collections, were maintained on YPDA agar plates (1% yeast extract, 2% Bacto-peptone, 2% Dextrose, 0.002% Adenine, 2% agar) at room temperature or stored at −80°C in 15% glycerol. We verified the gene disruption/deletion by PCR and Sanger sequencing. For stationary phase, cells from a single colony were inoculated into 20 ml liquid YPDA (1% yeast extract, 2% Bacto-peptone, 2% Dextrose, 0.002% Adenine) and incubated for 5 days at 200 rpm, 30°C. On day 5, 3.5 ml from each sample was added to fresh media, pre-warmed RPMI or RPMI + 10% HI-FBS, to a total volume of 100 ml, and incubated at 37°C with 60 rpm shaking. Samples were collected at 30 min, 2 hours, and 4 hours. Two biological replicates were performed for each of the four strains. RPMI 1640 with L-glutamine, sodium bicarbonate and pH indicator phenol red (Sigma R8758) and heat-inactivated serum (HI-FBS; Sigma F9665, Lot# BCCB5091 ) was aliquoted and stored at -20°C until required.

**RNA extraction and library preparation**

Briefly, *C. neoformans* cultures were fixed in methanol and dry ice, lyophilized, lysed by bead-beating in denaturing RLT buffer or TRI reagent, and RNA extracted using the Qiagen RNeasy Plant Mini Kit (Qiagen, Valencia, CA, USA). RNA-seq dataset 1 (reactivation) libraries were prepared with the RNATag-Seq protocol with rRNA depletion [(69)](https://paperpile.com/c/bq2XYM/WQlL) and dataset 2 (*GAT201* dependence) with QuantSeq FWD 3’ mRNA-Seq Library Prep Kit (Lexogen, Vienna, Austria). Full details are in the appended materials and methods.

*RNA-seq Dataset 1*

At each time point, 15 ml of the 100 ml culture (1 ml of stationary phase culture) was collected and immediately fixed in 7 ml Methanol (32% v/v final) in a 50 mL falcon tube on dry ice. Fixed cells were pelleted, transferred to a 1.5 ml screw-top tube in 1 mL ddH2O, and pelleted again. Pellets were lyophilized and mixed with 200 µL zirconium beads and ground on a bead-beater (Biospec 112011EUR) for 5 minutes. 1400 µL of buffer RLT (Qiagen) + 1% β-mercaptoethanol was added, and the mixture vortexed. RNA was then extracted by the Qiagen RNeasy Plant Mini Kit according to the manufacturer’s protocol for filamentous fungi, proceeding from step 4. RNA quality was checked by nanodrop and agilent bioanalyzer.

*RNA-seq Dataset 2*

At each time point, 15 ml of culture (2 ml of stationary phase culture) was collected and immediately fixed in 7 ml Methanol (32% v/v final) in a 50 mL falcon tube on dry ice. Fixed cells were pelleted, transferred to a 1.5 ml screw-top tube in 1 mL ddH2O, and pelleted again. Pellets were lyophilized, then mixed with 200 µL zirconium beads + 1 ml TRI reagent (Invitrogen) and incubated at room temperature for 5 mins followed by flash freezing in a dry ice/ethanol bath. Samples were thawed at room temperature and subjected to mechanical lysis by bead beating (using a PreCellys machine) for 3 x 10 seconds (6000 rpm) followed by a pause of 20 seconds and placed on ice for 1 minute. This was repeated 10 times. After mechanical disruption the zirconium beads were pelleted and the supernatant transferred to a QIA shredder spin column from the Qiagen RNeasy Plant Mini Kit (Qiagen, Valencia, CA, USA) and RNA was then extracted according to the manufacturer’s protocol, proceeding from step 4. Total RNA quantity and quality were assessed using nanodrop and the Fragment Analyser Automated Capillary Electrophoresis System (Agilent Technologies Inc, #5300) and the Standard Sensitivity RNA Analysis Kit, 15nt (#DNF-471).

For the experiments in Fig S1 (Dataset 1), 2 µg of RNA from each sample was used as input for RNA sequencing by the RNATagSeq protocol [(69)](https://paperpile.com/c/bq2XYM/WQlL), with minor modifications including ribosomal RNA depleted using the Yeast RiboZero Gold kit (Illumina; now discontinued), and a random barcode added to the 2nd ligation primer, with 12 cycles of PCR. Libraries were sequenced on a Nextseq500 (Illumina).

For the experiments in Fig S6 (Dataset 2) 500 ng of RNA from each sample was used as input for cDNA library preparation using the QuantSeq FWD 3’ mRNA-Seq Library Prep Kit (Lexogen, Vienna, Austria) for Illumina platforms according to the manufacturer’s instructions. We spiked in 10 ng of *Saccharomyces cerevisiae* total RNA as a loading control, but did not use this spike-in information in the data analysis presented here. The QuantSeq protocol generates only one fragment per transcript, close to the 3’ end of the transcripts. cDNA fragments of ∼300 bp were purified from each library and confirmed for quality by the Fragment Analyser Automated Capillary Electrophoresis System (Agilent Technologies Inc, #5300) and the Standard Sensitivity NGS 1-6000bp Kit (#DNF-473-33). Single read sequencing was performed using the NextSeq 500/550 High-Output v2.5 (75 cycles) Kit (#20024906) on the NextSeq 550 platform (Illumina Inc, #SY-415-1002). Libraries were combined in a single equimolar pool of 56 based on Qubit and Bioanalyser assay results and run across a High-Output v2.5 Flow Cell.

**Supplementary References**

Numbering is continuous from main references.

89. Steen BR, Lian T, Zuyderduyn S, MacDonald WK, Marra M, Jones SJM, Kronstad JW. 2002. Temperature-regulated transcription in the pathogenic fungus Cryptococcus neoformans. Genome Res 12:1386–1400. https://doi.org/10.1101/gr.80202

90. Missall TA, Pusateri ME, Lodge JK. 2004. Thiol peroxidase is critical for virulence and resistance to nitric oxide and peroxide in the fungal pathogen, Cryptococcus neoformans. Mol Microbiol 51:1447–1458. https://doi.org/10.1111/j.1365-2958.2004.03921.x

91. Zaragoza O, Fries BC, Casadevall A. 2003. Induction of capsule growth in Cryptococcus neoformans by mammalian serum and CO(2). Infect Immun 71:6155–6164. https://doi.org/10.1128/IAI.71.11.6155-6164.2003
